# Exploring the Occurrence of Organic Contaminants in Human Semen through an Innovative LC-HRMS-Based Methodology Suitable for Target and Nontarget Analysis

**DOI:** 10.1021/acs.est.3c04347

**Published:** 2023-11-07

**Authors:** Elena Sánchez-Resino, Montse Marquès, Daniel Gutiérrez-Martín, Esteban Restrepo-Montes, María Ángeles Martínez, Albert Salas-Huetos, Nancy Babio, Jordi Salas-Salvadó, Rubén Gil-Solsona, Pablo Gago-Ferrero

**Affiliations:** † Laboratory of Toxicology and Environmental Health, School of Medicine, Universitat Rovira i Virgili, IISPV, Sant LLorenç 21, Reus, Catalonia 43201, Spain; ‡ Center of Environmental, Food and Toxicological Technology - TecnATox, Universitat Rovira i Virgili, Reus 43201, Spain; § Department of Environmental Chemistry, Institute of Environmental Assessment and Water Research − Severo Ochoa Excellence Center (IDAEA), Spanish Council of Scientific Research (CSIC), Barcelona 08034, Spain; ∥ Institute of Sustainable Processes (ISP) and Department of Analytical Chemistry, Faculty of Sciences, University of Valladolid (UVa), Valladolid 47011, Spain; ⊥ Departament de Bioquímica i Biotecnologia, Grup ANut-DSM, Institut d’Investigació Sanitària Pere Virgili, CIBEROBN, Fisiopatologia de la Obesidad y Nutrición (ISCIII), Universitat Rovira i Virgili, Reus 43201, Spain; # Departament de Ciències Mèdiques Bàsiques, Unitat de Medicina Preventiva, Grup ANut-DSM, Institut d’Investigació Sanitària Pere Virgili, CIBEROBN, Fisiopatologia de la Obesidad y Nutrición (ISCIII), Universitat Rovira i Virgili, Reus 43201, Spain; ∇ Department of Nutrition, Harvard T.H. Chan School of Public Health, Harvard University, Boston, Massachusetts 02115, United States

**Keywords:** high-resolution mass spectrometry, seminal plasma, human exposure, male fertility, semen quality, emerging pollutants, LED-FERTYL study

## Abstract

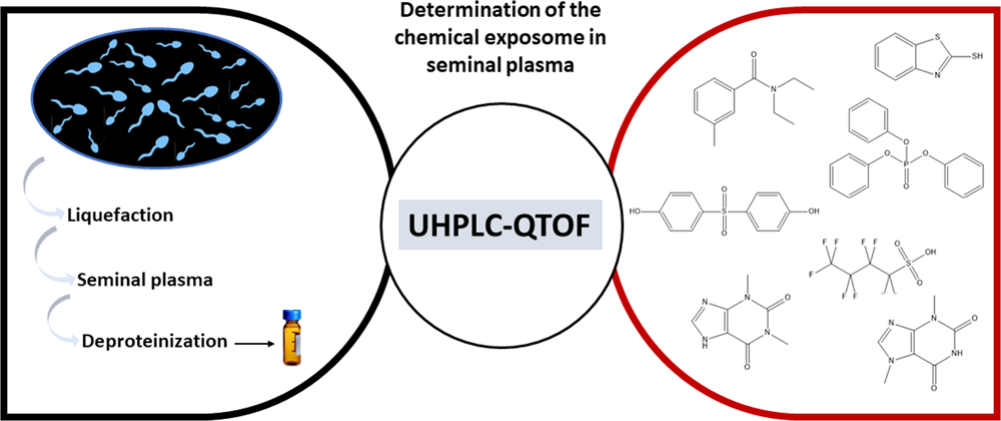

Understanding the potential impact of organic contaminants on male fertility is crucial, yet limited studies have examined these chemicals in semen, with most focusing on urine and blood. To address this gap, we developed and validated a robust LC-HRMS methodology for semen analysis, with a focus on polar and semipolar chemicals. Our methodology enables the quantitative (or semiquantitative) analysis of >2000 chemicals being compatible with suspect and nontarget strategies and providing unprecedented insights into the occurrence and potential bioaccumulation of diverse contaminants in this matrix. We comprehensively analyzed exogenous organic chemicals and associated metabolites in ten semen samples from Spanish participants collected in an area with a large presence of the chemical industry included in the LED-FERTYL Spanish study cohort. This investigation revealed the presence of various contaminants in semen, including plastic additives, PFAS, flame retardants, surfactants, and insecticides. Notably, prevalent plastic additives such as phthalic acid esters and bisphenols were identified, indicating potential health risks. Additionally, we uncovered previously understudied chemicals like the tire additive 2-mercaptobenzothiazole and specific organophosphate flame retardants. This study showcases the potential of our methodology as a valuable tool for large-scale cohort studies, providing insights into the association between contaminant exposure and the risk of male fertility impairments.

## Abstract 2

Evidence suggests male infertility is related to organic contaminant exposure, yet studies are limited. We developed a comprehensive HRMS methodology for polar and semipolar chemicals, analyzing semen samples, and identifying novel contaminants.

## 1 Introduction

Modern societies use an increasing number of chemicals, which are an integral part of modern life. Although much of the progress in recent decades is largely based on the manufacture of these compounds, it has become evident that their use has undesired environmental- and health-related secondary effects. Due to their physicochemical and biological properties, those chemicals, as well as their related metabolites and/or transformation products, may be persistent in the environment, bioaccumulative, and toxic.^1^ A robust body of evidence has convincingly linked chemical exposure to several adverse health outcomes.^2^ These effects include allergies,^3^ various types of cancer,^4^ neurological disorders,^5^ as well as reproductive health disorders.^6^

In recent decades, particularly during the past few years, there has been a significant decrease in the quality of semen in healthy men.^7^ One major reason for the observed defects in male reproductive function might be related to exposure to environmental contaminants,^8−10^ including trace elements as well as organic chemicals. Exposure to specific endocrine-disrupting trace elements such as As, Cd, Hg, Pb, Se, and Zn has been correlated with impaired male reproductive function, like defects in sperm functionality and low semen quality.^11^ However, assessing exposure to organic contaminants presents a more complex challenge due to the vast number of chemicals encountered in daily environments, including workplaces.^12^ An urgent research priority is to identify the specific organic chemicals (or chemical mixtures) that contribute to declining semen quality. In 2020, Kortenkamp et al. conducted a study that sheds light on this issue.^13^ They identified various molecular initiating events and linked effector chains that come together at nodal points, leading to shared downstream pathways and adverse outcomes. This study suggested a set of organic chemicals that should be considered in studies linking exposures to environmental contaminants and adverse effects in male reproductive function including (I) androgen receptor antagonists (e.g., bisphenols, parabens, azole chemicals, and polybrominated diphenyl ethers), (II) disruptors of prostaglandin signaling and InsL3 production (e.g., analgesics), (III) suppressors of testosterone synthesis (e.g., phthalates, acrylamide), (IV) inhibitors of steroidogenic enzymes (linuron), and (V) aryl hydrocarbon receptors agonists (e.g., polychlorinated dibenzodioxins). Besides, there are other additional studies suggesting that per- and polyfluoroalkyl substances (PFAS) affect semen quality^14^, as well as a large number of chemicals yet to be determined.

One of the primary gaps in this research field is that the majority of studies investigating the link between semen quality (mostly, sperm concentration and total sperm counts) and exposure to organic contaminants in human biofluids have focused on measuring chemical concentrations in only blood or urine samples. Specifically, researchers have primarily examined endocrine disruptor chemicals (EDCs) such as PFAS, organochlorine pesticides, phthalates, polychlorinated biphenyls (PCBs), polybrominated diphenyl ethers, and polybrominated biphenyls in these types of matrices.^15−17^ However, the analysis of these chemicals in semen remains infrequent, with only a few studies that have mainly investigated the presence of PFAS, phthalic acid esters (PAEs), pesticides, PCBs, and volatile organic compounds (VOCs) in seminal plasma. To provide a comprehensive summary of the studies conducted in semen, Table 1 outlines the analyzed chemicals, the number of samples tested, sample treatment and instrumental analysis, the range of concentrations, and limits of detection (LODs) when available.

**Table 1:** Summary of the Studies That Have Determined Organic Chemicals in Semen Samples in the Literature

chemical class	samples (*n*)^a^	sample treatment	instrum. analysis	chemicals analyzed (DF%^b^/concentration range)^c^	LOD^d^	year	ref
**organophosphates**	*n* = 123	steam distillation and LLE	NCI^e^	tris(1,3-dichloro-2-propyl)-phosphate (27%/5–50)	NA	1981	(21)
**pesticides, polychlorinated biphenyls (PCBs), and dioxines**	*n* = 191	LLE	LC-MS/MS	chemicals analyzed: NNIs^f^ (i.e., IMI, ACE, CLO, THM, DNT, THCP, NTP, FLO, SUL, IMIT) and their metabolites (i.e., DM-ACE, IMI-olefin, DM-CLO, DM-THM, and 5-OH-IMI)	DM-ACE (0.005), DM-CLO, and IMI-olefin (0.0005)	2022	(18)
				chemicals detected: DM-ACE (98%/≤0.422), IMI-olefin (86%/≤0.063), and DM-CLO (71%/≤0.232)			
	*n* = 21	NA	GC/MS	36 organochlorine pesticides and 35 PCBs; chemicals detected: mirex (100%/7–1455), methoxychlor (9%/NA), oxychlordane (9%/NA), and tetrachlorobenzene (4%/NA)	NA	2002	(22)
	*n* = 100	LLE and cleanup with a Florisil column	GLC	samples were categorized in 2 groups, named as fertile (*n* = 50, samples from fertile men) and infertile (*n* = 50, samples from infertile men); data is expressed as mean concentration (MC) in the format: (MC_fertile_/MC_infertile_)	NA	2007	(23)
				HCH isomers (α, β, γ, δ) (18/93); aldrin (0.3/0.2); and DDT and its metabolites (pp′DDT, opDDT, pp′DDE, pp′DDD) (23/45)			
	*n* = 174	LLE, derivatization, and sonication	LC-MS/MS	samples were categorized in 4 groups based on infertility: healthy (*n* = 84), slightly (*n* = 56), moderate (*n* = 20), and severely (*n* = 14)	NA	2015	(24)
				chemical analyzed: BPA; group (mean concentration): healthy (0.06); slightly (0.1); moderate (0.1); and severely (0.1)			
	*n* = 191	LLE, derivatization, and sonication	LC-MS/MS	samples were categorized in 4 groups based on infertility: healthy (*n* = 89), slightly (*n* = 59), moderate (*n* = 25), and severely (*n* = 18)	NA	2016	(25)
				chemical analyzed: BPA. Group (mean concentration): healthy (0.07); slightly (0.1); moderate (0.1); and severely (0.1)			
	*n* = 50	LLE and cleaned on a Florisil column	GLC	HCH isomers (α, β, γ, δ) (100%/1.28–4862); aldrin (6%/≤6.0); α-endosulfan (6%/≤82.99); pp′-DDT (nd); op′-DDT (nd); pp′-DDE (70%/≤830); and pp′-DDD (16%/≤5)	HCH isomers (0.10–0.30); aldrin (0.30); α-endosulfan (0.50); and DDT metabolites (0.30–0.50).	2000	(26)
	*n* = 50	grounded, extraction, and clean up	GLC	units: μg/g; HCB (34%/0.001–0.001), α-HCH (62%/0.001–0.006), β-HCH (8%/0.001–0.005), γ-HCH (70%/0.001–0.026), δ-HCH (72%/0.001–0.028), ε-HCH (62%/0.001–0.018), DDE (40%/0.001–0.012), op′DDT (nd), pp′DDT (nd), and DMDT (nd)	units: pg/g; HCB, BHC, and DDE (0.001); op’DDT (0.002), pp’DDT (0.004), and DMDT (0.010)	1981	(27)
	*n* = 16	Florisil column	GC-ECD	PCBs congeners 138, 153, and 180. MC: 11.7 ng/g	NA	1989	(28)
	*n* = 89	LLE and cleanup with Florisil	GC-ECD	chemicals analyzed: HCB, α-HCH, DDT, and metabolites, dieldrin, and PBCs	NA	2009	(29)
				chemical (DF%/MC): clophenA40 (14%/146), α-HCH (12%/6), HCB (31%/4.3), 2.4′-DDE (6%/6), 4,4′-DDE (20%/4), dieldrin (1%/1.4), 2,4-DDT (16%/14.6), and 4.4′- DDT (2%/7)			
	*n* = 217	LLE, cleanup, and gel chromatography	GC/HRMS	samples were categorized in 3 groups: (1) normospermia, *n* = 63, (2) pathospermia *n* = 105, and (3) fertile, *n* = 49	NA	2015	(30)
				chemical (MC for (3)/(1)/(2) in pg/g_lipids_): TCDD (19/31/58), PnCDD (28/59/47), HxDD (18/16/22), HpDD + OCDD (13/38/33), TCDF + PnCDF (98/294/279), HxCDF (22/16/19), and HpCDF + OCDF (11/9.3/21)			
	*n* = 17	EPA Method 8290	HRGC-HRMS	chemicals analyzed: dioxins, dibenzofurans, and the dioxin-like PCBs	NA	1996	(31)
				chemical (MC in ppq, ww): TCDD (3), PCDD (4), total HxCDD (38), HpCDD (89), OCDD (787), total PCDDs (920), TCDF (1), 1,2,3,7,8-PeCDF (1), 2,3,4,7,8-PeCDF (3), total HxCDF (13), total HpCDF (6), total OCDF (177), total PCDFs (201), and total PCDD/PCDFs (1,1)			
**per- and polyfluoroalkyl substances (PFAS)**	*n* = 651	ion-pair extraction	LC-MS/MS	PFOA (100%/0.04–2.9), PFNA (95%/≤0.03), PFOS (99%/≤9.9), and 6:2 Cl-PFESA (100%/0.01–1.37), PFBA (NA), PFPeA (NA), and PFHxA (NA)	LOQs range (ng/mL): 0.002–0.10	2020	(32)
	*n* = 664	ion-par extraction	LC-MS/MS	PFHpA (2%/≤0.060), PFOA (100%/0.04–2.966), PFNA (83%/≤0.36), PFDA (83%/≤0.25), PFUnDA (84%/≤0.214), PFDoDA (29%/≤0.062), PFTriDA (76%/≤0.09), PFTeDA (14%/≤0.060), PFBS (6%/≤0.094), PFHxS (31%/≤0.246), PFOS (96%/≤8.716), 6:2 Cl-PFESA (100%/0.005–1.368), 8:2 Cl-PFESA (31%/≤0.077), PFBA (NA), PFPeA (NA), and PFHxA (NA)	LOQs range (ng/mL): 0.002–0.10	2019	(33)
	*n* = 100	LLE	LC-HRMS	PFOA (96%/≤5.3) and PFOS (86%/≤1.1)	NA	2019	(34)
	*n* = 103	freeze-dry and extracted by ASE-SPE	LC-MS/MS	chemical (DF%/MC): PFBA (100%/3.9), PFOA (100%/0.9), PFPrA (100%/1.6), PFHxA (100%/4.3), PFPeA (100%/2.1), PFOS (100%/5.3), PFHpA (100%/0.5), PFHS (93%/0.13), and PFBS (94%/0.11)	PFOS (0.0162), PFOA (0.0137), PFHS (0.0188), PFHpA (0.0011), PFHxA (0.0037), PFBS (0.0127), PFPeA (0.0241), PFBA (0.0251), and PFPrA (0.0248)	2018	(35)
	*n* = 252	SPE	LC-MS/MS	PFOS (59%/≤5.4) and PFOA (2%/≤1.7)	NA	2012	(36)
	*n* = 59	LLE and nylon filtration (0.2 μm)	LC-MS/MS	seminal plasma; PFOS and/or PFAS in a 15% of samples; MC: PFOS (5.3); and PFOA (7.68)	unit ng/g fw: PFOS (1.5) and PFOA^3^	2015	(37)
				sperm cells; chemicals were nondetected (nd)			
**phthalic acid esters (PAEs)**	*n* = 687	phosphoric acid, deconjugation, and SPE	LC-MS/MS	chemical (DF%/MC in μg/L): MMP (35%/5.8), MEP (67%/2.3), MBP (99%/1.2), MBzP (29%/0.09), MEHP (100%/2.2), MEHHP (100%/0.2), MEOHP (79%/0.05), and MOP (13%/0.03)	LOD range (ng/mL): 0.0080–0.043	2016	(38)
	*n* = 52	LLE	LC-UV	DEP (0.1–1.3), DBP (0.09–0.5), and DEHP (0.08–0.9); units: mg/L	NA	2006	(39)
	*n* = 103	freeze-dry, incubated (β-glucuronidase), and ASE	LC-MS/MS	chemicals analyzed: MEHP; MnOP; MBzP; MHP; MBP; MiBP; MEP; MMP; ∑8m-PAE*s* = 2.1. MEHP (DF > 60%, media*n* = 0.5); MnOP (DF > 60%, media*n* = 0.6); MMP, MBP, MiBP, MEHP, and MnOP were frequently detected (DR% > 60); MBzP and MEP only detected in a few samples	LOD range (ng/mL): 0.0018–0.012	2020	(40)
	*n* = 99	LLE	LC-MS/MS	DEHP (97%/≤3215), MEHP (97%/≤5015), DBP (15%/≤6.3), MBP (96%/<268), and PA (96%/≤1762)	DEHP (3.5), MEHP (2.0), DBP (1.5), MBP (1.0), and PA (1.3)	2009	(41)
	*n* = 300	LLE	LC-UV	samples were categorized in 4 groups: (1) fertile rural, *n* = 40; (2) fertile urban, *n* = 60; (3) infertile rural, *n* = 88; and (4) infertile urban, *n* = 112	NA	2008	(42)
				chemical (MC of (1)/(2), (3)/(4) in μg/mL): DEP (0.6/0.7, 1.1/3.1), DBP (0.1/0.6, 1.1/1.6), DEHP (0.1/0.1, 0.33/0.7), DMP (0.3/0.5, 0.9/1.9), and DOP (0.04/0.2, 0.2/0.1)			
	*n* = 50	LLE	LC-MS/MS	chemical (MC in μg/mL): DEP (0.9), DBP (0.9), and DEHP (0.5)	1 ng/mL	2014	(43)
	*n* = 53	charcoal-dextran treatment, LLE, and acid cleanup	LC-UV–vis	samples were categorized in 2 groups: infertile, *n* = 21 and controls, *n* = 32; chemicals analyzed: 8 PCBs and 6 phthalates	LOD for PCBs range (ng/mL): 3.23–14,970	2002	(44)
				∑PCBs: infertile (7.6), controls (nd); ∑phthalates (DMP; DEP; DBP; BBP; DEHP; and DOP): infertile (2), controls (0.06); units: μg/mL			
	*n* = 290	incubation (β-glucuronidase) and SPE	LC-MS/MS	samples were categorized in 3 groups: (1) fertile, *n* = 37; (2) subfertile normal SQP, *n* = 124; and (3) subfertile abnormal SQP, *n* = 129	MMP (0.11), MEP (0.17), MiBP (0.18), MBP (0.13), MBzP (0.11), MEHP (0.12), MEHHP (0.18), MEOHP (0.17), MECPP (0.18), MINP (0.19), and MIDP (0.11)	2017	(45)
				chemical (MC for (1)/(2)/(3)): MMP (0.1/0.2/0.2), MEP (0.2/0.3/0.4), MiBP (0.4/0.4/0.4), MnBP (0.9/0.9/0.9), MBzP (0.1/0.1/0.1), MEHP (0.3/0.4/0.4), MEHHP (0.2/0.2/0.3), MEOHP (0.1/0.1/0.1), MECPP (0.2/0.2/0.3), MiNP (0.2/0.2/0.2), and MiDP (0.2/0.2/0.2)			
	*n* = 463	incubation (β-glucuronidase) and SPE	LC-MS/MS	MEP (30%/≤11.1), MBP (40%/≤9.6), MiBP (16%/≤4.9), MBzP (18%/≤7.9), MEHP (25%/≤12.3), MEHHP (5%/≤2.8), MEOHP (5%/≤1.2), MECPP (1%/≤0.4), MiNP (12%/≤1.7), MHiNP (nd), MOiNP (nd), and MCiOP (1%/≤0.2)	LOD range (ng/mL): 0.19–1.01	2010	(46)
	*n* = 60	incubation (β-glucuronidase) and SPE	LC-MS/MS	MMP (38%/≤265), MEP (68%/≤54), MBP (99%/≤44), MEHP (100%/0.21–223), MEHHP (100%/0.27–9.7), MEOHP (78%/≤2.29), MBzP (6%/NA), and MOP (6%/NA)	LOD range (ng/mL): 0.008– 0–043	2015	(47)
**volatile organic compounds (VOCs)**	*n* = 57	HS-SPME	GC/MS and GC/sensor	chemicals analyzed (nonquantified): sulfenyl compounds, primary alcohols, organonitrogen compounds, organobromides, ketones, hydrazones, heteroaromatic compounds, ethers, esters, carboxylic acids, benzene and substituted derivatives, amines, amides, alkenes, alkanes, aldehydes, and acetamides	NA	2019	(48)
	*n* = 33	HS-SPME	GC/MS	chemicals analyzed (nonquantified): 2-anthracenamine, acetone, *n*-hexane, butanal, D-limonene, pentanal, and pyrrole	NA	2022	(49)
	*n* = 69	HS-SPME	GC/MS	total number of identified VOCs: 196; nonquantified	NA	2018	(50)

a *n*: number of samples analyzed.

b Detection frequency (DF%): number of positive samples from the total, expressed as a percentage.

c Chemicals analyzed are expressed in this column. For those detected, DF% and concentration range are included in the following format: chemical (DF%/min–max). If the minimum value of the range corresponded with nondetected, it was simplified with the format: Chemical (DF%/≤max). In some cases, mean concentration (MC) substitutes the concentration range (min–max), so the format is Chemical (DF%/MC). If other formats were applied, it was directly indicated. Concentration units were ng/mL unless stated otherwise.

d Limit of detection (LOD) expressed in ng/mL, unless otherwise stated. In some cases, LODs or limits of quantification (LOQs) are expressed as a range, and it is directly indicated.

e Abbreviations: accelerated solvent extraction (ASE), electron capture detection (ECD), electrospray ionization negative mode (ESI(−)), electrospray ionization positive mode (ESI(+)), fresh weight (fw), gas chromatography (GC), gas–liquid chromatography (GLC), headspace-solid phase microextraction (HS-SPME), high-resolution gas chromatography and high-resolution mass spectroscopy (HRGC-HRMS), instrumental analysis (Instrum. analysis), kidney-Yang Deficiency syndrome (KYDS), limit of detection (LOD), limit of quantification (LOQ), liquid chromatography (LC), liquid–liquid extraction (LLE), mean concentration (MC), negative chemical ionization (NCI), nonavailable (NA), nondetected (nd), parts per quadrillion (ppq), references (ref), semen quality parameters (SQP), solid phase extraction (SPE), and wet-weight (ww).

f Abbreviations of chemicals: 1,1-dichloro-2,2-bis(p-chlorophenyl)ethane (pp′DDD); 2,2′,3,4,4′,5,5′-heptachlorobiphenyl (PCB congener 180); 2,2′,3,4,4′,5′-hexachlorobipheniyl (PCB congener 138); 2,2′,4,4′,5,5′-hexachlorobipheniyl (PCB congener 153), 5-hidroxy-imidalocoprid (5-OH-IMI), 6:2 chlorinated polyfluorinated ether sulfonate (6:2 Cl-PFESAs), 8:2 chlorinated polyfluorinated ether sulfonate (8:2 Cl-PFESAs), acetamiprid (ACE), benzyl phthalate (BBP), bisphenol A (BPA), butyl mono-*n*-butyl phthalate (MnBP), clothianidin (CLO), desmethyl-acetamiprid (DM-ACE), desmethyl-clothianidin (DM-CLO), desmethyl-thiamethoxam (DM-THM), dichloro-2,2-bis(*p*-chlorophenyl ethylene (pp′DDE), dichlorodiphenyltrichloroethane (DDT), diethylhexyl phthalate (DEHP), diethyl phthalate (DEP), dimethyl phthalate (DMP), di-*n*-butyl phthalate (DBP), dinoctyl phthalate(DOP), dinotefuran (DNT), flonicamid (FLO), heptachlordibenzofuran (HpCDF), heptachlordibenzo-*p*-dioxin (HpDD), hexachlordibenzo-*p*-dioxin (HxDD), hexachlordibenzofuran (HxCDF), hexachlorobenzene (HCB), hexachlorocyclohexane (HCH), imidacloprid (IMI), imidacloprid-olefin (IMI-olefin), imidaclothiz (IMIT), methoxychlor (DMDT), mono(2-carboxymethyl) hexyl phthalate (MECPP), mono(2-ethyl-5-hydroxyhexyl) phthalate (MEHHP), mono(2-ethyl-5-oxohexyl) phthalate (MEOHP), mono(2-ethylhexyl) phthalate (MEHP), monobenzyl phthalate (MBzP), monoethyl phthalate (MEP), monohexyl phthalate (MHP), monoiso-butyl phthalate (MiBP), monoiso-decyl phthalate (MiDP), monoiso-nonyl phthalate (MiNP), monomethyl phthalate (MMP), mono-*n*-butyl phthalate (MBP), mono-*n*-octyl phthalate (MnOP), mono-*n*-octyl phthalate (MOP), neonicotinoid insecticides (NNIs), nitenpyram (NTP), nonafluorovaleric acid (PFPeA), octachlordibenzofuran (OCDF), octachlordibenzo-*p*-dioxin (OCDD), perchloropentacyclodecane (Mirex), pentachlordibenzofuran (PnCDF), pentachlordibenzo-*p*-dioxin (PnCDD), pentafluoropropionic acid (PFPrA), perfluorobutanesulfonic acid (PFBS), perfluorobutyric acid (PFBA), perfluoroctanoic acid (PFOA), perfluorodecanoate (PFDA), perfluorododecanoate (PFDoDA), perfluoroheptanoic acid (PFHpA), perfluorohexanesulfonate (PFHS), perfluorohexanesulfonate (PFHxS), perfluorononanoate (PFNA), perfluorooctanesulfonic acid (PFOS), perfluorotetradecanoate (PFTeDA), perfluorotridecanoate (PFTriDA), perfluoroundecanoate (PFUnDA), phthalic acid (PA), polychlorinated biphenyls (PCBs), sulfoxaflor (SUL), tetrachlordibenzo-*p*-dioxin (TCDD), thiacloprid (THCP), thiamethoxam (THM), and undecafluorohexanoic acid (PFHxA).

It is apparent that a more comprehensive understanding of the link between chemical exposure and the decrease in male fertility can be achieved by analyzing the exogenous chemical profiles and related metabolites in human semen. Compared to other biofluid analyses, examining semen may provide more specific information on the possible adverse effects occurring in the testes.^18^ However, the lack of effective methodologies for detecting a broad range of organic chemicals in semen is concerning, especially for polar and semipolar compounds, which are known to have adverse effects.^19^ It is essential to have powerful analytical methods that can take advantage of the latest capabilities to detect these chemicals in complex matrices, such as semen. Fortunately, recent advances in high-resolution mass spectrometry (HRMS) have significantly improved analytical capabilities for detecting trace-level chemicals in environmental and biological samples. These advancements have enabled a shift from detecting a few compounds in a targeted manner to high-sensitivity methodologies capable of capturing a large number of substances through a combination of target, suspect, and nontarget strategies.^20^ This progress has opened the door to gaining insight into which compounds bioaccumulate or pseudobioaccumulate in humans and conducting further epidemiological studies on their association with specific adverse health outcomes. The aims of this study were (I) to establish and validate a robust methodology using liquid chromatography (LC) coupled with HRMS for the quantitative determination of a broad spectrum of organic chemicals (>2000), while ensuring its suitability for direct application in both suspect and nontarget studies; and (II) to determine the profiles of exogenous organic chemicals and associated metabolites in ten human semen samples obtained from the LED-FERTYL Spanish study cohort, including healthy men aged 18–40 year old. The purpose includes illustrating how the methodology could be of significant value in assessing large cohorts and advancing the study of the association between exposure to contaminants and male fertility impairments.

## 2 Materials and Methods

### 2.1 Study Population and Sample Collection

Semen samples of participants were randomly selected and retrieved from the ongoing LED-FERTYL (lifestyle and environmental determinants of seminogram and other male fertility-related parameters) study. The recruitment began in February 2021 and included healthy Spanish volunteers between 18 and 40 years old from the general population. Participants were contacted via telephone or email to be informed of the steps to follow during their participation in the study. Lifestyle, including diet, physical activities, sedentary behaviors, smoking, alcohol consumption, and general demographic characteristics, were collected using questionnaires. Afterward, participants were appointed at Hospital Universitari Sant Joan de Reus, Reus, Tarragona (Spain), where anthropometric parameters were assessed along with the collection of biological samples. For the present study, we selected a cohort subset of participants residing in areas with a significant presence of the chemical industry. Collection and examination of semen samples followed the standards set by the World Health Organization (2010).^51^ Briefly, participants were provided with specific instructions to ensure the purity of the semen samples. First, they were advised to abstain from sexual activity for 3–7 days before collection. Second, before sample collection, they were instructed to wash their hands thoroughly. They were then directed to collect the sample directly into a polypropylene wide-mouthed container in a private room using masturbation, ensuring to avoid any hand contact with the semen itself. Despite these clear instructions, we couldn't guarantee the complete adherence of all participants. At this stage, procedural blanks were also collected, and semen was substituted with Milli-Q water. Then, the semen samples were liquefied at 37 °C for 30 min and analyzed using a computer-assisted sperm analysis (CASA) system (SCA, Microptic). The parameters analyzed include semen volume, pH, sperm concentration, total count, total motility, sperm vitality, and sperm morphology. An aliquot of 150 μL of semen was stored at −80 °C until chemical analysis. Further details of the samples are described in Table 2. Study participants provided written informed consent, and the Institut d’Investigació Sanitària Pere Virgili has approved the protocol.

**Table 2:** Characteristics of Study Participants and Semen Samples

sample	age (years)	weight (kg)	height (cm)	BMI^a^ (kg/m^2^)	tobacco smoking	alcohol consuming (g/day)	seminal quality parameters^b^						
							abstinence period^c^ (days)	pH	volume (mL)	concentration of spermatozoa(×10^6^/mL)	vitality (%)	total motility (progressive + nonprogressive) (%)	normal form (%)
S1	29	64	174	21.1	no	0	3	8.5	7.0	41.2	71.5	69.0	10.5
S2	23	78.3	187	22.4	no	3.95	3	8.5	4.5	91.5	83.5	78.5	16.5
S3	32	74.5	175	24.1	no	20.23	3	8.5	4.0	154.7	70.0	54.0	15.0
S4	40	68.5	169	24	no	20.94	4	8.5	4.5	227.2	86.5	59.6	13.5
S5	36	75.5	177	24	no	2.72	4	8.5	6.2	69.5	80.0	59.3	19.0
S6	32	115	182	34.7	no	31.58	5	8.5	4.0	206.5	73.0	65.0	17.5
S7	29	75	185	21.9	no	5.28	6	8.5	5.5	125.2	76.5	74.0	7.5
S8	31	90	177	28.8	FS^d^	0.68	7	8.5	4.0	198.7	83.0	71.5	7.0
S9	36	75	175	24.5	FS^d^	20.19	6	8.5	3.2	219.5	91.0	56.5	8.0
S10	37	90.5	170	31.2	yes	2.03	3	8.5	2.3	52.0	80.0	73.7	21.3

a Body mass index (BMI), calculated as weight/(height^2^) in kg/m^2^.

b Calculated according to World Health Organization (WHO Laboratory Manual for the Examination and Processing of Human Semen 2010).

c Abstinence period: period of abstinence of ejaculation until sample collection.

d Former smoker.

### 2.2 Chemicals, Reagents, and Analyte Selection

This methodology aims to develop a comprehensive approach for wide-scope screening, encompassing a variety of analytes with a wide range of physicochemical properties. Out of the >2000 chemicals included in the target methodology, as published elsewhere,^52^ a validation data set was selected to ensure representativeness, considering the chromatographic retention time (RT), the ionization mode, and the physicochemical properties (log *K*_ow_). A total of 91 chemicals were selected and covered an RT range between 3 and 14 min in +ESI and between 4 and 12 min in −ESI. Regarding the ionization mode, 39 chemicals ionize more effectively in positive, other 39 in negative, and the rest were ionized in both modes. Log *K*_ow_ covered a wide range between −0.3 to 6.4. Thus, the validation set contained 91 diverse chemicals making up approximately 5% of the database (see Supplementary SI-1, Table S1) and including pharmaceutically active compounds (PhACs), biocides, food additives, personal care products (PCPs), natural products, or industrial products (such as plasticizers, perfluorinated compounds, UV-filters, or flame retardants). Further information about reagents, analytical and internal standards (ISs, 24 labeled chemicals in total) are present in SI-1, Tables S1 and S2. Both analytical standards and ISs were obtained from LGC (Teddington, UK).

### 2.3 Sample Treatment

A simple sample treatment was selected and fully validated, focusing on mitigating the loss of analytes with different physicochemical properties. The objective was to enable comprehensive screening and combine target, suspect, and nontarget approaches. Semen samples were thawed at room temperature, and an internal standard (clothianidin-d3) was added as a surrogate at 50 ng/mL (in-vial concentration) in each sample and incubated in a water bath at 37 °C until liquefaction (15 min). Then, an aliquot of 150 μL was centrifuged (10,000*g*, 10 min) to obtain seminal plasma. Supernatant (100 μL) was then mixed with ACN (300 μL) for protein precipitation and further centrifuged (10,000*g*, 10 min). Supernatant was then transferred to a chromatographic vial and stored at −80 °C until analysis. Before injection, a mixture of 24 internal standards (see Supplementary SI-1, Table S2) was added in each sample to control instrumental variations in response. Another sample treatment including preconcentration was proposed but discarded, as discussed in the section Sample Treatment Optimization under Results and Discussion.

### 2.4 UPLC-QTOF Acquisition and Data Analysis for Applicability

An ultrahigh-performance liquid chromatography (UHPLC) system with a Bruker Elute Pump HPG 1300 coupled to a QTOF Impact II (Bruker, Bremen, Germany) was used for the analysis. The chromatographic separation was performed on an Intensity Solo column (2.1 × 100 mm, 1.8 μm) from Bruker, preceded by a guard column, CORTECTS C18, 1.7 μm 2.1 × 5 mm from Waters (Milford, USA), thermostated in an air oven at 40 °C. Instrument was operated in broadband collision-induced dissociation (bbCID), a data-independent acquisition (DIA) mode, at 3 scans per second. The aqueous mobile phase consisted of H2O:MeOH (99:1) with additives: 5 mM ammonium formate and 0.01% formic acid for ESI(+), and 5 mM ammonium acetate for (−)ESI. The organic phase was MeOH with identical additives for each ionization mode, respectively. Details on the instrumental analysis can be found in SI-2.

### 2.5 Method Validation and Quantitative Analysis

The method was validated in terms of accuracy, precision, matrix effect, sensitivity, linearity, and linear range using a validation set of 91 chemicals with a wide range of physicochemical properties (Table S1). A pooled semen sample (*n* = 5) was utilized for validation, and residual levels within this pool were taken into consideration (see SI-3). Accuracy was calculated as absolute recovery using five replicates at three concentration levels (1, 5, and 20 ng/mL in extract) (see SI-3). The linearity of the methodology was assessed by using matrix-matched calibration curves at nine different points (ranging from 0.05 to 100 ng/mL). The linear range was defined as the interval between the limit of quantification and the maximum point of the matrix-matched calibration curve that remained within the acceptable range of linearity. Precision was calculated as the repeatability of the method in terms of the relative standard deviation using the same standard injected by quintuplicate. The matrix effect was determined as the peak area intensity of the chemicals in matrix-matched calibration (at 10 ng/mL) compared to the peak area observed in the solvent calibration curve (at 10 ng/mL), expressed as a percentage. Matrix effect values above 100% indicate an enhancement effect, while those below 100% signify a suppression effect. Sensitivity was estimated with the limit of quantification (LOQ), considered the lowest point of the matrix-matched calibration curve with a peak width of more than 7 points and a signal-to-noise ratio higher than 10. More information about the validation process is provided in SI-3. For data processing, TASQ 2.1 software (Bruker Daltonics, Bremen, Germany) was used. Quantitative results were calculated by using matrix-matched calibration curves.

It is necessary to clarify that the methodology enables the targeted analysis of 2316 chemicals, as precise RT along with clear mass spectra have been acquired for all these chemicals under the described experimental conditions. Once the screening is conducted, a number of chemicals are detected in a targeted manner (with experimental RT and mass spectra), some of which may be included in the validation set or not. For chemicals not included, it is necessary to prepare an additional matrix-matched calibration curve and conduct further recovery experiments, followed by injecting the samples into the LC-HRMS system. This approach allows for the acquisition of reliable quantitative results for all chemicals. If one wishes to avoid this final step or if obtaining the standard for certain compounds is not feasible, semiquantitative results can be obtained using a model for ionization efficiency (IE) based on a quantitative structure-ionization relationship model (QSIR). The specifics of QSIR model development and validation workflow can be found elsewhere.^53^

### 2.6 Quality Assurance and Quality Control (QA/QC)

We implemented a comprehensive set of quality assurance and quality control (QA/QC) protocols throughout all analytical stages in the laboratory. In order to maintain a clean working environment, we cleaned the work surface with water and acetone at the beginning of each day and baked all of the glassware at 450 °C prior to use. To account for potential contamination during the analytical process, we conducted the analysis of procedural blanks (*n* = 3). In these blanks, semen samples were replaced with an equal volume of HPLC-grade water, which was then subjected to the same processing steps as those applied to the real semen samples. The concentration of these blanks plus three times the standard deviation were deducted from the concentration of the actual samples for each respective chemical. Methanol and a standard mixture (50 nG/mL) were regularly injected (every 20 samples) to assess instrument carryover and stability. The compound clothianidin-d3 was used as a surrogate to control potential sample treatment losses and instrument performance. Any deviation in the surrogate’s performance between samples would signify a proportionate loss of all chemicals attributable to the sample treatment process. For the quantification of the analytes, the IS mixture was added prior to instrumental analysis to correct for the instrumental analysis variations. The instrument was internally calibrated using sodium formate/acetate in 2-propanol/water (50:50) with 0.2% acid for each analysis, which was also stored at the first 30 s of each sample run. In addition, the instrument was externally calibrated with a sodium formate solution before each sequence.

## 3 Results and Discussion

### 3.1 Sample Treatment Optimization

HRMS-based target methodologies have gained popularity in recent years due to their unbiased approach, allowing for the screening of various chemicals present in a sample without predefining them. However, compared to classical target analysis, HRMS-based strategies have lower sensitivity, making it necessary to create sample treatment strategies that are as simple as possible but have a maximum concentration factor. Different extraction methods, including liquid–liquid extraction, SPE protocols, and deconjugation steps, have been described in the literature and are extensively discussed in Table 1. However, these methods are specific to a particular chemical class. Since our goal was to cover as many chemicals as possible in a single analysis, the method should focus on avoiding potential losses more than on the recovery of specific chemical families. For this reason, despite hypothesizing that a simple deproteinization may be the best option, we wanted to test whether the use of a preconcentration step will benefit LODs in semen samples.

The deproteinization protocol, named Method 1, involved a deproteinization via precipitation with ACN followed by centrifugation and LC-HRMS analysis as explained in the Sample Treatment section. This method, similar to those normally used for blood plasma in metabolomic studies, may be a suitable strategy for exposomics analyses as it reduces potential chemical losses. Due to the nature of the method, the sample ends up being diluted. However, since some exogenous chemicals may be present at very low levels, we tested whether adding a preconcentration step could improve LODs in real samples using an already validated SPE methodology (named Method 2), which has given acceptable results in other matrices such as wastewater,^52^ biota,^54^ or placenta.^55^ Method 2 was analogous to Method 1, but the supernatant was collected after protein precipitation (see SI-4). A preconcentration factor of 1× was achieved for this method. In contrast, Method 1 dilutes the original sample four times, thus assuming a preconcentration factor of ×0.25. Preconcentration in biofluids is limited due to the small volumes typically available (normally range from μL to a few mL), making it challenging to achieve high preconcentration factors. Also, matrix effects may even worsen LODs when trying to concentrate human samples, making preconcentration less effective in this context.

We tested both Method 1 and Method 2 in terms of recovery, limits of quantification, and precision. The performance of the two extraction methods was compared using a set of chemicals with diverse physicochemical properties, including pesticides, PhACs, plastic additives, nicotine, or tire additives, among others. The comparison was based on triplicate measurements of 5 ng/mL of the analytes in extract. Method 1 showed better recovery values (ranging from 24 to 125%, with 60% falling between 70 and 130%) than Method 2 (which ranged from 11 to 85%). Additionally, Method 1 exhibited better repeatability (with a median of 18%) than Method 2 (with a median of 30%). Notably, Method 1 achieved better limits of quantification, with 90% of the values below 0.5 ng/mL, compared with only 50% for Method 2. It is worth mentioning that including an additional SPE step in the protocol complicates the analysis, increases the time required, and introduces the possibility of sample contamination. Based on these results, Method 1 was deemed simpler, faster, and more effective, and we therefore conducted a more comprehensive validation of this method, which was slightly diluted but still produced favorable extraction outcomes.

### 3.2 Method Validation

**Figure 1:**
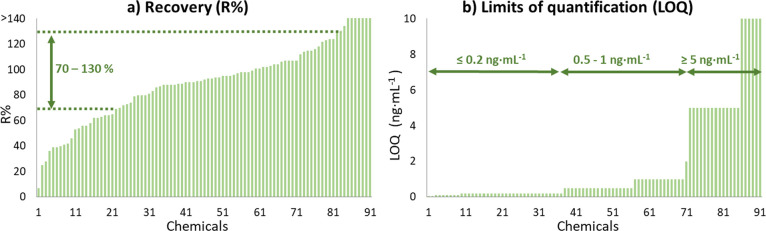
Method validation parameters. (a) Recoveries at 20 ng/mL and (b) limits of quantification.

The selected method, consisting of a deproteinization step followed by centrifugation, was validated with the set of 91 chemicals shown in SI-1, Table S1, but at three different concentration levels. The details on the performance of the method are summarized in Figure 1 and SI-5, Table S3.

As depicted in Figure 1a, recovery values were good for the majority of chemicals, with 66% exhibiting values between 70 and 130%, while only tetradecylamine showed a poor recovery (<20%). Despite the simplicity of the method, it is possible that some chemicals were lost during the protein precipitation step. However, most of the chemicals were well preserved in the sample for subsequent analysis. The precision of the method was found to be acceptable, with 67% of chemicals exhibiting a relative standard deviation below 25% (see SI-4, Table S3). Additionally, linearity was observed to be higher than 0.99 for 67% of the evaluated chemicals and higher than 0.98 for the rest of the chemicals except for tetradecylamine (*R*^2^ = 0.974), nicotine (*R*^2^ = 0.979), and alachlor (*R*^2^ = 0.975). The matrix effect was good for the simplicity of the sample treatment, despite the difficulty of avoiding these effects in ESI-LC analysis, especially for biological samples with high content of endogenous substances.^56,57^ A 43% of the chemicals showed signal suppression (ME% < 100%) while a 56% exhibited enhancement (ME% > 100%). Only 18 chemicals exhibited a strong signal suppression, with a matrix effect below 50% (see SI-4, Table S3).

In terms of sensitivity, all chemicals were quantifiable in the extract at levels of ≤10, and 0.5 ng/mL was the median LOQ for the 91 chemicals (SI-5, Table S3). In addition, 69 of these chemicals were quantifiable at ≤1 ng/mL and 36 at levels even ≤0.2 ng/mL. The high sensitivity of the method is essential for human biomonitoring, as these chemicals may cause negative effects even at these low levels. Previous human biomonitoring studies showed similar LOQs in other biofluids, for example: 0.1–0.6 ng/mL for 4 metabolites of organophosphate flame retardants (OPFRs) in urine by LC-HRMS;^58^ or 0.04/0.052 ng/mL median values in serum/urine for 24 EDCs by LC/MS.^59^ Thus, this study provided satisfactory results, especially considering the wide number and variety of chemicals assessed. In addition, the simplicity of the methodology allows the implementation of this methodology in large human biomonitoring cohorts, which is an actual research gap in semen biofluid.

### 3.3 Wide-Scope Screening of Polar and Semipolar Organic Chemicals in Samples of Human Seminal Plasma

**Figure 2:**
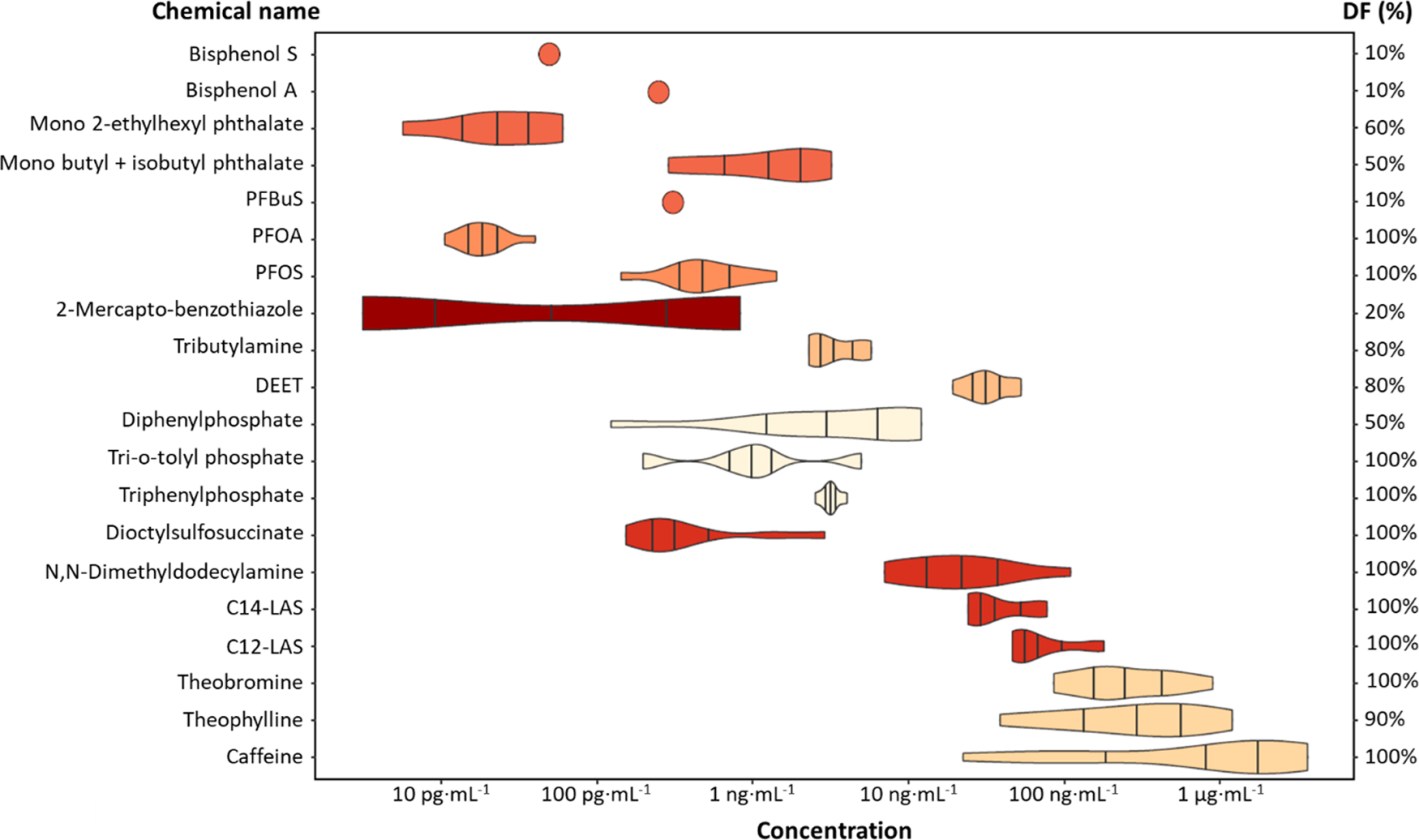
Concentration and detection frequency (DF%) of polar and semipolar organic chemicals in samples of human seminal plasma. The violin plot represents the distribution of the DF of 20 polar and semipolar organic chemicals detected in samples of human seminal plasma (*n* = 10) against their concentration. All the analytes were identified above the LOD. Vertical bars inside each violin represent the quartiles, while filled circles represent compounds that have a 10% DF.

A wide variety of chemicals were detected in the ten selected semen samples, including plastic additives and tire additives, PFAS, flame retardants, surfactants, insecticides, and food additives. In total, 21 chemicals were determined, with concentrations ranging from pg/mL to a few μg/mL. Of these chemicals, 14 were present in more than 50% of the samples. For chemicals not included in the validation, a matrix-matched calibration curve and additional recovery experiments were conducted. The results are illustrated in the form of a violin plot in Figure 2, and details on RT, compound class, *m*/z, identification points (IP), detection frequency (DF), and concentration range are summarized in Table 3. The IP system was applied to convey the confidence level of the results in Table 3, as described by Alygizakis et al.^110^ The complete information regarding the concentration of each chemical in the samples, along with a chromatogram, is available in Supplementary SI-6, Table S4 and Figure S1.

**Table 3:**
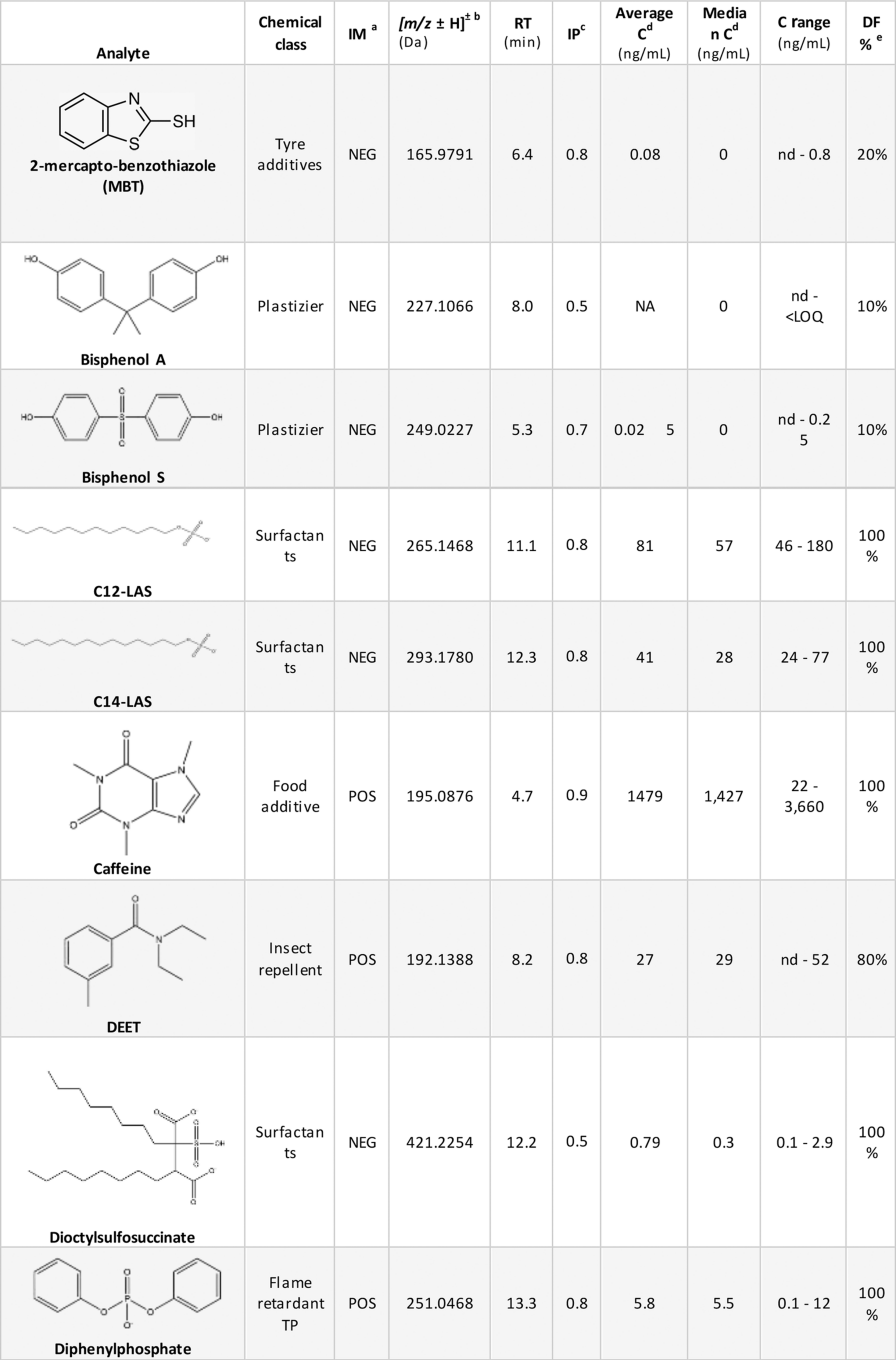

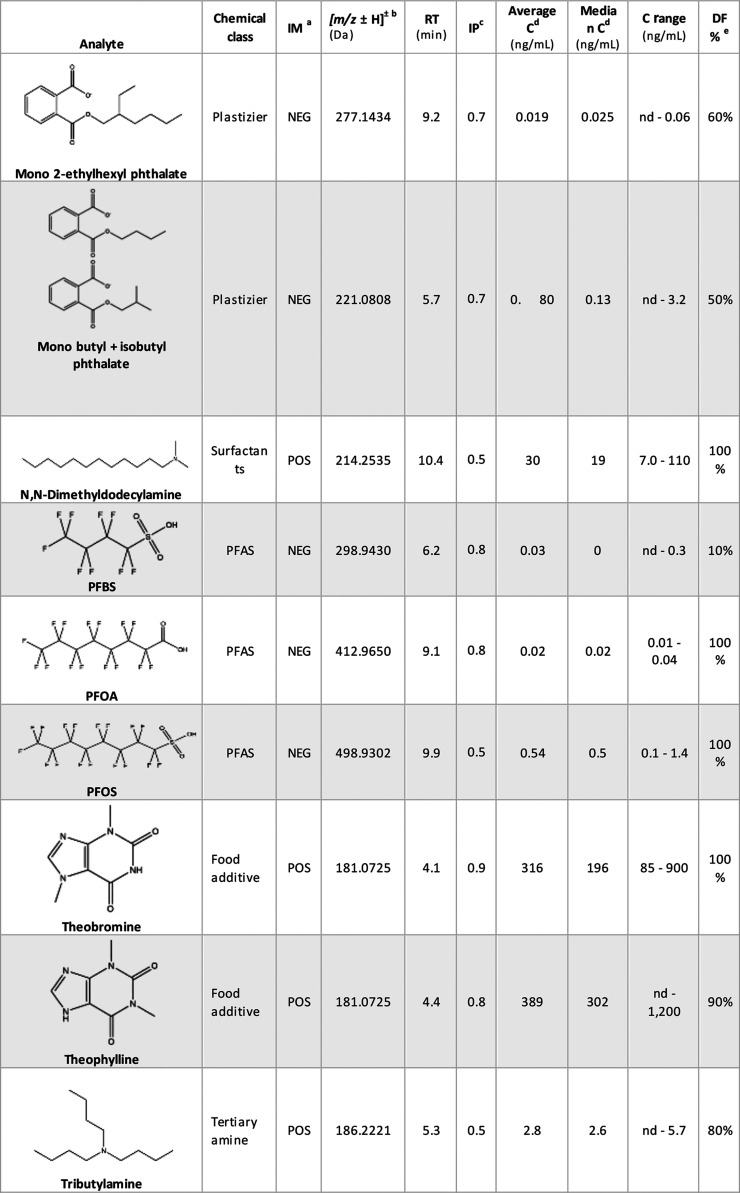

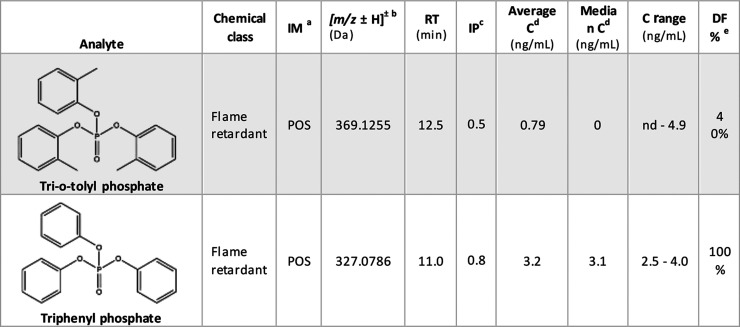
Results of Organic Chemicals Identified in Samples of Human Seminal Plasma

a Ionization mode (IM).

b *m*/*z* for the ionized chemical in positive IM (POS) or in negative IM (NEG).

c Identification points (IP): values represent the confidence level for compound identification, with a score closer to 1 indicating higher certainty. Derived from the scoring system proposed by Alygizakis et al.^110^

d To calculate the average and median, values <LOQ were considered LOQ/2.

e Detection frequency expressed in percentage (DF%). Abbreviations: perfluorobutanesulfonic acid (PFBS), per- and polyfluoroalkyl substances (PFAS), perfluorooctanoic acid (PFOA), perfluorooctanesulfonate (PFOS), diethyltoluamide (DEET), transformation product (TP), dodecyl sulfate (C12-LAS), and tetradecyl sulfate (C14-LAS).

#### 3.3.1 Plastic Additives

Five plastic additives were present in the analyzed samples, including three PAEs and two bisphenols. These chemicals are currently receiving significant attention from regulatory agencies such as the European Chemicals Agency (ECHA) due to concerns about their potential impact on health.^60^ Mono 2-ethylhexyl phthalate (MEHP) and the mixture of mono butyl (MBP) + isobutyl (MiBP) phthalate were found with a DF of 60% (concentrations up to 0.06 ng/mL) and DF: 50% (concentrations up to 3.2 ng/mL in the detected samples), respectively. These chemicals are commonly used as plasticizers in polyvinyl chloride (PVC products), such as pipes and tubing, as well as in food packaging.^61^ Bisphenol S (BPS) and Bisphenol A (BPA) were also detected in one of the samples each at 0.05 and 0.25 ng/mL, respectively. These chemicals are used to produce polymers and resins, so they can be found in everyday consumer items such as reusable plastic tableware, drink bottles, and food packaging, as well as in thermal paper or textiles.^62^ The presence of phthalates and BPA in human semen has already been reported in previous literature reports, as described in Table 1. In those studies, phthalates showed high DF%, up to 100%, with similar concentrations as those here reported.^39,41,42,46−48^ Regarding BPA, mean concentrations ranged from 0.66 to 0.179 ng/mL in two previous studies related to human infertility.^24,25^ Thus far, there are no existing data regarding the incidence of BPS in semen. Exposure to these chemicals has been associated mainly with oral intake (e.g., dietary ingestion) but also dermal contact with plastic products or inhalation of aerosols. Studies have monitored their presence in other biofluids such as urine or blood.^63,64^ Despite being present in low concentrations, these chemicals have been linked to numerous health issues including endocrine disruption, genotoxicity, cytotoxicity, reproductive toxicity, dioxin-like effects, and neurotoxicity.^64−67^ Specifically, bisphenols and PAEs have been identified as chemicals whose monitoring is essential in studying adverse effects on male reproductive function.^13^ Thus, these findings provide new insights into the occurrence of these chemicals in human semen, further highlighting their potential impact on reproductive health.

#### 3.3.2 PFAS

Three PFAS, including perfluorooctanesulfonic acid (PFOS), perfluorooctanoic acid (PFOA), and perfluorobutanesulfonic acid (PFBS), were detected in the semen samples. PFOS and PFOA are the most produced and studied PFAS,^68^ which is consistent with their 100% DF in these semen samples. PFOS and PFOA were detected in the range 0.14–1.42 and 0.01 to 0.04 ng/mL, respectively. PFOS was found at similar levels than previous studies, while PFOA was generally found at slightly lower concentrations (Table 1). Out of the samples tested, only one showed a positive result for PFBS, with a concentration of 0.31 ng/mL. This level of detection was also consistent with previous studies conducted on over 600 samples from the Reproductive Medical Center (Nanjing Jinling Hospital, China), which had a <6.5% DF (LOQ: 0.004 ng/mL, concentration range: LOQ—0.094 ng/mL).^32,33^ However, this finding differs from Son et al.’s study, which reported a DF of 94% out of the 103 semen samples analyzed, with an LOQ of 0.0381 ng/mL and a mean concentration of 0.11 ng/mL.^35^ Several exposition pathways have been proposed for these widely used chemicals, including ingestion of polluted drinking water and food, inhalation, or dermal contact with contaminated media.^68,69,70^ In fact, drinking water health advisory limit have changed from 70 ng/L (combining both PFOS and PFOA) in 2016 to 0.004 and 0.02 ng/L for PFOA and PFOS, respectively, in 2022.^71^ Adverse health effects derived from the exposure to PFAS include cancer,^72^ as well as immune, metabolic, and neurodevelopmental effects.^69^ Petersen et al. conducted a review of the epidemiological evidence regarding the potential relationship between exposure to PFAS and male reproductive health issues including semen quality, reproductive hormones, cryptorchidism, hypospadias, and testicular cancer. However, the results were inconclusive.^14^ The direct analysis of PFAS in semen may give a better correlation with semen quality parameters than the common blood analysis, as mentioned by Son et al.^35^ Thus, the methodology developed in this study may aid in future health assessments related to reproductive health.

The case of 2-mercaptobenzothiazole (MBT) is of particular interest as it is primarily used in the rubber industry and was found in 20% of the samples, with concentrations up to 0.83 ng/mL. MBT has not been previously reported in semen samples, and there is limited knowledge about its presence in human biofluids, with only a few studies conducted on urine.^73,74^ The main exposure route is likely through inhalation, as it is present in airborne particulate matter due to its widespread use as a tire additive.^75^ Dermal contact is also a possible route of exposure, as MBT is present in daily rubber products (e.g., detected in rubber mulch).^76^ However, MBT was absent from the 236 retail food samples analyzed by Barnes et al. indicating that food ingestion might not be a significant route of exposure.^77^ MBT has been associated with adverse human health outcomes, such as skin conditions (e.g., dermatitis^78^ and cancer (IARC) classified it as probably carcinogenic to humans—Group 2A).^78^ Overall, there is limited knowledge about the exposure and health impact of this chemical.

The samples analyzed revealed the presence of three OPFRs, substances added to many products during the manufacturing process to reduce the risk and propagation of fire.^79^ Specifically, triphenyl phosphate (TPhP) and its metabolite, diphenylphosphate (DPhP), were detected in all samples, while tri-*o*-tolyl phosphate (TOTP) was present in half of them. The concentrations ranged as follows: TPhP (2.52–4.03 ng/mL), DPhP (0.12–13.3 ng/mL), and TOTP (nd–4.97 ng/mL). To our knowledge, only one study from 1980 has reported the presence of a flame retardant, tris(1,3-dichloro-2-propyl)-phosphate, in semen samples at a concentration range of 5–50 ng/mL.^21^ However, biomonitoring studies in urine, hair, nails, human milk, or blood^80−85^ have demonstrated their ubiquity in the human body. Human exposure to flame retardants occurs through inhalation, ingestion, and dermal contact.^79,86−88^ However, little is known about their impact on human health. As reviewed by Chupeau et al.,^79^ these chemicals may have an impact on reproductive functions (e.g., TPhP is inversely associated with the success of in vitro fertilization,^89^ decreased sperm concentration,^90^ or slightly increased gestational duration^91^), as well as thyroid systems, neurodevelopmental functions (e.g., TPhP decreased intellectual quotient,^92^ respiratory system and immunotoxicity, or dermal effects^92^). However, more research is needed to properly assess the human risk posed by these OPFRs.

Four additional surfactants were also detected, all of them with a DF: 100%. These chemicals (and their concentration range) are dioctylsulfosuccinate (DOSS) (0.15–1.33 ng/mL), *N*,*N*-dimethyldodecylamine (7.02–110 ng/mL), dodecyl sulfate (C12-LAS) 46.53–180 ng/mL), and tetradecyl sulfate (C14-LAS) (24.1–77.8 ng/mL). To the best of our knowledge, none of these four chemicals have been reported in human semen samples. Surfactants are commonly added to home and personal care products, but also in several industries such as textile, polymers, paints and coating, leather, printing, agriculture, or even in food products.^93−95^ Regarding these chemicals, little is known about their human exposure or toxicity, so their detection in these semen samples may provide new insight into the assessment of their toxicity and relevance.

Diethyltoluamide (DEET), a common active ingredient in insect repellents, was found in 80% of the samples, with levels up to 52.9 ng/mL in the detected samples. The large use of this chemical has resulted in its frequent detection in the human body (e.g., urine and serum).^96,97^ However, no previous information regarding its presence in human semen has been found until now. DEET is typically applied to the skin, which is the main route of exposure, and it is associated with low levels of acute toxicity when absorbed through the skin, ingested orally, or inhaled.^98^ The scientific literature contains only a small number of studies that describe adverse effects and incidents of human poisoning associated with DEET, such as case reports for dermal reactions^99^ and some neurological effects.^100^ Chronic toxicity and oncogenicity, developmental toxicity, neurotoxicity, and reproductive toxicity have also been evaluated for DEET, mainly in animal research, but its use is generally considered safe.^98^

Tributylamine (TrBA) is reported for the first time in semen, found with a DF of 80%, with concentrations up to 5.77 ng/mL. This tertiary amine have applications in different areas including lubricating materials, corrosion inhibitors, textiles, paints, dyes, and the pharmaceutical industry.^101^ Humans are mainly exposed via the respiratory tract and through dermal contact, producing membrane irritation and breathing difficulties.^102,103^ However, studies are mainly focused on animals; therefore, little is known about the effects on human health.

Several food additives, namely, the xanthine alkaloids theobromine, theophylline, and caffeine, were detected in the samples (DF: 100%, range: 85.9–900 ng/mL for theobromine, DF: 90%, range: nd–1200 ng/mL for theophylline, and DF: 100%, range: 22.4–3112 ng/mL for caffeine). These are well-known chemicals found in human biofluids such as urine, blood, or human milk,^104,105^ and are considered safe with several health benefits. However, some results suggest a potential association between these chemicals and reproductive issues. Caffeine intake has been controversially associated with various adverse pregnancy outcomes and negative impacts on men’s fertility,^106,107^ while theobromine was classified as category 1B in reproductive toxicity by the Japan Chemical Management Center (NITE-CMC) for producing adverse effects in animals.^108^ On the other hand, the addition of theophylline to freeze semen samples for assisted reproductive technology was proposed and resulted in better preservation of sperm motility.^109^

### 3.4 Implications and Limitations

Although studies in this area are scarce, evidence suggests that male infertility is associated with exposure to organic exogenous chemicals. It is particularly relevant that there is a lack of studies carried out involving the chemical analysis of semen samples. In this study, we aimed to address the existing knowledge gap by developing and validating a robust LC-HRMS methodology for the analysis of semen samples, allowing for the comprehensive quantitative analysis of over 2000 chemicals. While this study primarily emphasized wide-scope target screening to achieve our objectives, this methodology is also fully compatible with suspect and nontarget approaches, thereby increasing the coverage of chemical compounds and their metabolites. It has the potential to provide a novel understanding of contaminants in semen, with potential bioaccumulation influenced by spermatogenesis and the seminal plasma’s lipid profile. However, like any methodology, it has limitations in terms of the chemical space covered. The described method focuses on polar and semipolar chemicals that are ionizable by electrospray. In order to obtain a more comprehensive picture of the chemicals present in this type of sample, it would be necessary to combine our method with other chromatographic techniques (e.g., GC-HRMS) and alternative ionization methods (e.g., APCI). The method employs a nonspecific sample treatment to encompass a large number of substances. While this is one of its main strengths, it may result in lower recoveries for some chemicals (which can end up in a slight underestimation of concentrations using semiquantitative approaches) and LODs for certain substances (e.g., PFAS) compared to targeted methodologies that focus solely on specific compounds.

Through the analysis of ten semen samples from participants residing in an area with a significant presence of the chemical industry, we identified various contaminants, including plastic additives, PFAS, flame retardants, surfactants, and insecticides. Despite the origin of the samples, none of the detected chemicals can be directly linked to the chemical industry. The reduced number of samples may have influenced this finding. Of particular concern was the prevalent presence of plastic additives such as phthalic acid ester metabolites and bisphenols, which indicate potential health risks. The presence of previously understudied chemicals such as tire additives or specific OPFRs was also identified. It is also noteworthy that our results emphasize the importance of proper procedural blanks as a fundamental step to minimize the number of false positives. In these procedural blanks, contamination was detected for 8 chemicals (*N*,*N*-dimethyldodecylamine, C12-LAS, C14-LAS, mono-2-ethylhexyl phthalate, diphenyl phosphate, PFOS, PFOA, and PFBS). In these cases, we adjusted the concentration of the samples by subtracting the concentration of the blanks plus three times their standard deviation. Therefore, we only reported the concentration in samples where its level was significantly higher than that of the procedural blanks.

Overall, these findings highlight the potential value of our developed methodology as a valuable tool for large-scale cohort studies, enabling the assessment of contaminant exposure directly in semen and its association with the risk of male fertility impairments. However, it is important to remark that the analysis of only ten samples is a small and nonrepresentative number to draw solid conclusions, but it serves to demonstrate the importance of further investigations in this direction. Therefore, further research and epidemiological studies utilizing our methodology or similar approaches are warranted to elucidate the specific organic chemicals or chemical mixtures that contribute to male fertility impairments. Such studies may also promote intervention methods to reduce exposure to harmful chemicals. This will help deepen our understanding of the mechanisms underlying the impact of organic contaminants on male fertility and establish strategies for mitigating these effects.

## Supporting Information Available

Chemicals, reagents, and analyte selection; UPLC-QTOF acquisition and data analysis for applicability; method validation and quantitative analysis; sample treatment optimization; method validation; and wide-scope screening of polar and semipolar organic chemicals in samples of human seminal plasma (PDF)
