# Supplementary material for: Exploring the Occurrence of Organic Contaminants in Human Semen through an Innovative LC-HRMS-Based Methodology Suitable for Target and Nontarget Analysis
Source: Environ Sci Technol. 2023 Nov 7;57(48):19236–52. doi: 10.1021/acs.est.3c04347 (PMC10722465; doi:10.1021/acs.est.3c04347)
Supplement: Supplementary file 1 — es3c04347_si_001.pdf [file es3c04347_si_001.pdf]

Supplementary Information

Exploring the occurrence of organic contaminants in human semen through an innovative LC-HRMS-based methodology suitable for target and non-target analysis

Elena Sánchez-Resino[1], Montse Marquès[1,2], Daniel Gutiérrez-Martín[3,4], Esteban Restrepo-Montes[3], Maria Ángeles Martínez-Rodriguez[5], Albert Salas-Huetos[6,7], Nancy Babio[5], Jordi Salas-Salvadó[5], Rubén Gil-Solsona[3] and Pablo Gago-Ferrero[3*]

[1]Universitat Rovira I Virgili, Laboratory of Toxicology and Environmental Health, School of Medicine, IISPV, Sant LLorenç 21, 43201, Reus, Catalonia, Spain.
[2]Center of Environmental, Food and Toxicological Technology - TecnATox, Universitat Rovira i Virgili, Spain.
[3]Department of Environmental Chemistry, Institute of Environmental Assessment and Water Research – Severo Ochoa Excellence Center (IDAEA), Spanish Council of Scientific Research (CSIC), Barcelona 08034, Spain.
[4]Institute of Sustainable Processes (ISP) and Department of Analytical Chemistry, Faculty of Sciences, University of Valladolid (UVa), 47011 Valladolid, Spain.
[5]Universitat Rovira i Virgili. Departament de Bioquímica i Biotecnologia. Grup ANut-DSM. Institut d'Investigació Sanitària Pere Virgili. CIBEROBN. Fisiopatologia de la Obesidad y Nutrición (ISCIII).
[6]Universitat Rovira i Virgili. Departament de Ciències Mèdiques Bàsiques, Unitat de Medicina Preventiva. Grup ANut-DSM. Institut d'Investigació Sanitària Pere Virgili. CIBEROBN. Fisiopatologia de la Obesidad y Nutrición (ISCIII).
[7]Department of Nutrition, Harvard T.H. Chan School of Public Health, Harvard University, Boston, MA02115, USA.

*Corresponding author: pablo.gago@idaea.csic.es, +34 934006100

Number of supplementary pages: 13
Number of supplementary Figures: 1
Number of supplementary Tables: 4

Index

SI-1. Chemicals, reagents and analyte selection.

Chemicals and reagents. **Page S2**

Table S.1. List of analytical standards used for the validation. **Page S2 - S5**

Table S.2. List of internal standards used for the validation. **Page S5**

SI-2 UPLC-QTOF acquisition and data analysis for applicability

UPLC-QTOF acquisition parameters **Page S6**

SI-3. Method validation and quantitative analysis

Method validation parameters. Page S7

SI-4. Sample treatment optimization.

Sample preparation for Method 2. **Page S8**

SI-5. Method validation.

Table S.3. Performance of the method validation. **Page S9 - S12**

SI-6. Wide-scope screening of polar and semi-polar organic chemicals in samples of human seminal plasma.

Table S.4. Distribution of analyte concentrations in each sample. **Page S13**

Figure S.1. Chromatograms for all identified chemicals. **Page S14 - S32**

SI-1. Chemicals, reagents and analyte selection.

Chemicals and reagents

Standard solutions were prepared at 1000 ng/mL in methanol and further diluted in methanol until 1 ng/mL. All the solvents used were HPLC-grade. Acetonitrile (ACN), methanol (MeOH), water, formic acid (>99% purity), ammonium acetate and ammonium formate (≥99.0% purity) were purchased from Merck (Darmstadt, Germany). Milli-Q water was provided by a Milli-Q purification apparatus (Aurium, PRO-VFT, Sartorius, Göttingen, Germany). For the preconcentration step, discussed in section 3.1: ethyl acetate 99.6% from Acros Organics, ammonia solution 32% from Merck, solid phase extraction (SPE) empty cartridges, frits, Sepra ZT (30 µm, 85Å) powder, Sepra ZTL-WCX (100 µm, 300Å) powder and Sepra ZTL-WAX (115 µm, 330Å) powder were purchased from Phenomenex (Madrid, Spain), and Isolute ENV+ was purchased from Biotage (Uppsala, Sweeden).

Table S.1. List of analytical standards used for the validation.

Name	IM[a]	Formula	RT[b]	*m/z* parent[c]	m/z fragment[d]	LogKow[e]	Chemical class
**Tryptoline**	POS	C11H12N2	4.3	173.1073	144.0808	1.3	Natural product
**2-amino-Benzothiazole**	POS	C7H6N2S	6.0	151.0324	124.0215	1.9	Industrial product
**2-ethylhexyl 4-(dimethylamino)benzoate**	POS	C17H27NO2	14.1	278.2115	166.0862	5.0	UV-filter
**4-Aminoantipyrine**	POS	C11H13N3O	5.2	204.1131	146.0600	0.1	PhACs TP
**Alachlor**	POS	C14H20ClNO2	10.3	270.1255	162.1277	3.5	Herbicide
**Carbamazepine**	POS	C15H12N2O	7.3	237.1022	194.0966	2.1	PhAC
**Carbendazim**	POS	C9H9N3O2	5.5	192.0768	160.0507	1.5	Biocide
**Chlorpyriphos**	POS	C9H12Cl3NO3PS	12.9	349.9336	197.9275	5.3	Biocide
**Dimethomorph**	POS	C21H22ClNO4	9.5	388.1310	301.0639	3.9	Biocide
**Dimethyl benzotriazole**	POS	C8H9N3	6.6	148.0869	NA	1.8	Industrial product
**Diuron**	POS / NEG	C9H10Cl2N2O	8.3 / 8.2	233.0243 / 231.0097	72.0444 / 185.9519	2.9	Herbicide
**Drometrizole**	POS	C13H11N3O	5.1	226.0975	120.0555	2.6	Industrial product
**Enrofloxacin**	POS	C19H22FN3O3	4.75	360.1718	316.1820	0.6	PhAC
**Enzacamene**	POS	C18H22O	12.8	255.1743	212.1187	5.1	UV-filter
**Flumequine**	POS	C14H12FNO3	7.15	262.0874	244.0768	1.6	PhAC
**Lauryl diethanolamide**	POS	C16H33NO3	11.6	288.2533	106.0861	3.5	PCP
**Malathion**	POS	C10H19PS2O6	9.6	331.0433	99.0077	2.4	Biocide
**Metalaxyl**	POS	C15H21NO4	8.1	280.1543	192.1384	1.5	Biocide
**Methiocarb**	POS	C11H15NSO2	9.2	226.0896	121.0650	2.9	Biocide
**Methyl-benzotriazole**	POS / NEG	C7H7N3	5.9	134.0713 / 132.0567	NA / 104.0506	1.1	Industrial product
**Molinate**	POS	C9H17NSO	9.8	188.1104	126.0916	3.2	Herbicide

**Mono(5-carboxy-2-ethylpentyl) Phthalate**	POS / NEG	C16H20O6	7.5 / 4.8	309.1333 / 307.1187	149.0234 / 121.0295	2.5	Plastisizer TP
**Monocyclohexyl Phthalate**	POS / NEG	C14H16O4	8.5 / 6.5	249.1121 / 247.0976	149.0234 / 121.0295	2.9	Plastisizer TP
**Monopentyl Phthalate**	POS / NEG	C13H16O4	8.6 / 6.7	237.1121 / 235.0976	149.0234 / 121.0295	3.7	Plastisizer TP
**N-acetyl sulfadiazine**	POS / NEG	C12H12N4SO3	4.6 / 4.2	293.0703 / 291.0557	134.0602 / 227.0938	-0.2	PhAC TP
**N-acetyl sulfamethazine**	POS / NEG	C14H16N4O3S	4.9	321.1016 / 319.0870	204.0438 / 134.0611	0.1	PhAC TP
**N-acetyl sulfapyridine**	POS / NEG	C13H13N3O3S	4.6	292.0750 / 290.0605	134.0602 / NA	-0.1	PhAC TP
**Nalidixic acid**	POS	C12H12N2O3	6.9	233.0921	215.0816	1.4	PhAC
**N-desmethyl venlafaxine**	POS	C16H25NO2	5.9	264.1958	215.1430	3.0	PhAC TP
**Nicotine**	POS	C10H14N2	3.4	163.1230	132.0807	0.9	Smoke contaminant
**Oxadiazon**	POS	C15H18Cl2N2O3	12.5	345.0767	219.9567	4.8	Herbicide
**Oxolinic acid**	POS	C13H11NO5	6	262.0710	244.0604	0.9	PhAC
**Phenazone**	POS	C11H12N2O	5.1	189.1022	NA	1.2	PhAC
**Propanil**	POS	C9H9Cl2NO	9.1	218.0134	161.9869	3.1	Herbicide
**Sulfadiazine**	POS	C10H10N4O2S	5.6	251.0597	156.0114	-0.2	PhAC
**Sulfadimethoxine**	POS / NEG	C12H14N4O4S	4.5 / 5.1	311.0809 / 309.0663	156.0116 / 230.0809	1.1	PhAC
**Sulfamerazine**	POS	C11H12N4O2S	4.5	265.0754	156.0115	0.4	PhAC
**Sulfamethoxazole**	POS	C10H11N3O3S	4.9	254.0594	156.0114	0.9	PhAC
**Sulfamethoxypyridazine**	POS / NEG	C11H12N4O3S	4.7	281.0703 / 279.0557	156.0120	0.5	PhAC
**Sulfaquinoxaline**	POS / NEG	C14H12N4SO2	5.8 / 5.0	301.0754 / 299.0608	156.0114 / 144.0570	1.7	PhAC
**Sulfathiazole**	POS / NEG	C9H9N3O2S2	4.3 / 4.2	256.0209 / 254.0063	156.0120	0.9	PhAC
**Tris(2-chloroethyl) phosphate (TCEP)**	POS	C6H12Cl3PO4	6.9	284.9612	160.9767	1.3	Flame retardant
**Tris(2-chloropropyl) phosphate**	POS	C9H18Cl3PO4	9.3	327.0081	174.9908	2.6	Flame retardant
**Terbumeton**	POS	C10H19N5O	8.9	226.1662	170.1040	1.7	Herbicide
**Tetradecylamine**	POS	C14H31N	11.7	214.2529	57.0699	6.4	PCP
**Tonalide**	POS	C18H26O	13.6	259.2056	175.1120	5.3	PCP
**Triclocarban**	POS / NEG	C13H9Cl3N2O	11.7	314.9860 / 312.9708	161.9872 / 159.9723	5.3	Biocide
**Tri-o-tolyl phosphate**	POS	C21H21PO4	12.9	369.1250	91.0544	6.1	Flame retardant
**Tryptamine**	POS	C10H12N2	4.4	161.1073	144.0808	1.2	Natural product
**Umbelliferone**	POS	C9H6O3	5.3	163.0390	119.0491	1.9	Natural product
**Venlafaxine**	POS	C17H27NO2	5.9	278.2115	58.0654	2.7	PhAC

**Zoxamide**	POS	C14H16Cl3NO2	11.1	336.0319	186.9715	4.3	Biocide
**2,2’-Dihydroxy-4-methoxybenzophenone**	NEG	C14H12O4	9.2	243.0663	123.0452	3.2	UV-filter
**2-Benzothiazolesulfonic acid**	NEG	C7H4NNaO3S2	4.6	213.9638	134.0074	1.7	Industrial product
**Methyl 3,4-dihydroxybenzoate**	NEG	C8H8O4	5.2	167.0350	NA	1.5	PCP
**3,4-Dihydroxybenzoic Acid**	NEG	C7H6O4	6.0	153.0193	109.0295	0.6	PCP
**Benzyl paraben**	NEG	C14H12O3	9.2	227.0714	136.0166	1.7	Food additive
**Isopropyl paraben**	NEG	C7H6O3	7.7	179.0714	136.0166	1.6	Food additive
**Methyl paraben**	NEG	C8H8O3	6.0	151.0401	136.0166	2.2	Food additive
**4-Hydroxybenzophenone**	NEG	C13H10O2	7.5	197.0608	NA	3.4	UV-filter
**6:2 Fluorotelomer sulfonate**	NEG	C8H5F13O3S	9.1	426.9679	80.9652	3.9	Perfluorinated compound
**Benzophenone-1**	NEG	C13H10O3	5.9	213.0557	93.0345	2.9	UV-filter
**Benzophenone-2**	NEG	C13H10O5	6.2	245.0455	135.0089	2.1	UV-filter
**Benzophenone-3**	NEG	C14H12O3	9.1	227.0714	136.0165	3.4	UV-filter
**Benzophenone-4**	NEG	C14H12O6S	5.5	307.0282	211.0389	0.4	UV-filter
**Bisphenol AP**	NEG	C20H18O2	9.4	289.1234	274.0999	4.3	Plastisizer
**Bisphenol E**	NEG	C14H14O2	7.4	213.0921	NA	2.2	Plastisizer
**Bisphenol G**	NEG	C21H28O2	11.5	311.2017	NA	5.4	Plastisizer
**Bisphenol M**	NEG	C24H26O2	11.5	345.1860	NA	6.4	Plastisizer
**Bisphenol P**	NEG	C24H26O2	11.5	345.1860	315.1386	6.4	Plastisizer
**Bisphenol Z**	NEG	C18H20O2	9.9	267.1391	145.0659	4.8	Plastisizer
**Bisphenol A**	NEG	C15H16O2	8.0	227.1078	211.0770	3.8	Plastisizer
**Bisphenol AF**	NEG	C15H10F6O2	9.3	335.0512	265.0482	4.2	Plastisizer
**Bisphenol B**	NEG	C16H18O2	8.9	241.1234	211.0770	4.2	Plastisizer
**Bisphenol F**	NEG	C13H12O2	9.2	199.0765	93.0346	2.6	Plastisizer
**Bisphenol S**	NEG	C12H10O4S	5.3	249.0227	155.9886	1.9	Plastisizer
**Clarithromycin**	NEG	C38H69NO13	9.3	746.4696	NA	3.2	PhAC
**Di-1,2-Chloroethylphosphonic Acid**	NEG	C2H5Cl2O3P	4.4	220.9543	NA	-0.3	Plant hormone
**Ethyl 3,4-Dihydroxybenzoate**	NEG	C9H10O4	6.0	181.0506	NA	1.8	Food additive
**Gemfibrozil**	NEG	C15H22O3	10.5	249.1496	121.0646	3.6	PhAC
**Mecoprop**	NEG	C10H11ClO3	6.8	213.0324	141.0113	2.9	Herbicide
**Mono benzyl phthalate**	NEG	C15H12O4	5.9	255.0663	121.0295	2.9	Plastisizer TP
**Mono isobutyl phthalate**	NEG	C12H14O4	5.7	221.0819	121.0295	2.4	Plastisizer TP
**Mono methyl phthalate**	NEG	C9H8O4	4.0	179.0350	121.0295	1.5	Plastisizer TP
**Mono n-butyl phthalate**	NEG	C12H14O4	5.7	221.0819	121.0295	2.5	Plastisizer TP
**Mono(2-ethyl-5-hydroxyhexyl) Phthalate**	NEG	C16H22O5	6.2	293.1394	121.0295	2.7	Plastisizer TP
**Mono(2-ethyl-5-oxohexyl)phthalate**	NEG	C16H22O5	7.7	291.1238	121.0295	2.7	Plastisizer TP
**Mono-2-ethylhexyl phthalate**	NEG	C16H22O4	9.2	277.1445	121.0295	4.4	Plastisizer TP
**Monooctyl phthalate**	NEG	C16H22O4	9.6	277.1445	121.0295	4.4	Plastisizer TP
**O-desmethyl venlafaxine**	NEG	C16H25NO2	5.1	262.1813	230.0809	2.6	PhAC TP
**Methyl 3,4-dichlorophenylcarbamate (SWEP)**	NEG	C8H7Cl2NO2	9.4	217.9781	185.9522	3.1	Herbicide

[a]Prefereably ionization mode (IM): "POS" for positive IM, "NEG" for negative IM and "POS / NEG" when the chemical ionized in both modes. [b]Retention time (RT) for the prefereably ionization mode. When both POS and NEG were possible and presented a different RT, the RT is expressed as RTPOS / RTNEG. [c]*m/z* for the parent chemical in the indicated IM. When both POS and NEG were possible, the m/z is expressed as m/z_{POS} / $m/z_{NEG.}$ [d]*m/z* for the main fragment of the chemical in the indicated IM. When both POS and NEG were possibleand presented a different main fragment, the m/z is expressed as m/z_{POS} / $m/z_{NEG.}$ [e]*LogKow retrieved from Pubchem.*
Abbreviations. Non-available (NA), pharmaceutically active compound (PhAC), transformation products (TP), and personal care product (PCP)

Table S.2. *List of internal standards used for validation. Chlothianidin-d3 was used as a surrogate, and the rest of the IS were added just before LC-HRMS analysis.*

Name	Ionization mode	m/z	RT
**Nicotine-d3**	**POS**	166.1418	3.6
**Atenolol-d7**	**POS**	274.2142	3.9
**Methyl paraben-d3**	**POS / NEG**	157.0797 / 154.0589	4.2 / 6
**Metronidazole-d4**	**POS**	176.0967	4.3
**Caffeine-d3**	**POS**	198.1065	4.7
**Thiamethoxam-d3**	**POS**	295.0454	4.7
**Benzotriazole-d4**	**POS / NEG**	124.0807 / 122.0661	5 / 5.1
**Clothianidin-d3**	**POS / NEG**	253.0348 / 251.0202	5.1 / 5.1
**Citalopram-d4**	**POS**	329.1962	6
**Carbamazepine-d10**	**POS**	247.1651	7.1
**Dimethyl phthalate - d5**	**POS**	200.074	7.5
**Venlafaxine-d6**	**POS**	315.1736	7.8
**Diclofenac-d4 (phenyl-d4)**	**POS**	300.049	9.8
**Benzophenone-3 - d5 (BP3-d5)**	**POS**	234.1173	10.6
**Triphenylphosphate-d15**	**POS**	342.1722	10.8
**Bis(2-ethylhexyl) phthalate-d4**	**POS**	395.3093	14.9
**Acetamionophen-d4**	**NEG**	154.0811	4.2
**Bisphenol S - d8 (BPS-d8)**	**NEG**	257.0729	5.3
**2,6-Di-tert-butyl-4-methylphenol-d21**	**NEG**	240.3072	8.8
**Benzylparaben-d4**	**NEG**	231.0964	9.1
**Gemfibrozil-d6**	**NEG**	255.1872	10.5
**Octylphenol-d17**	**NEG**	222.2664	13
**Nonylphenol-d14**	**NEG**	223.2005	13.6
**Stearic acid-13C18**	**NEG**	301.3243	14.5

SI-2. UPLC-QTOF acquisition and data analysis for applicability

UPLC-QTOF acquisition parameters

The mobile phase composition for positive ionization mode (+ESI) was H_2O:MeOH (99:1) with 5 mM ammonium formate and 0.01% formic acid for the aqueous phase (A), and MeOH with 5 mM ammonium formate and 0.01 % formic acid for the organic phase (B). Mobile phase composition for negative ionization mode (-ESI) was H_2O:MeOH (99:1) with 5mM ammonium acetate for (A), and MeOH with 5 mM ammonium acetate for (B). The initial flow was 0.2 mL/min and changed during the sequence as follow (RT (min), flow (mL/min)): RT=2.5, flow 0.223; RT=14, flow 0.400; RT=16, flow 0.480; RT=19.10, flow 0.200. Total sequence time: 20min. The gradient was 96% A in initial conditions (0-0.99 min), 81.7% A (1-2.49 min), 50% A (2.5-13.99 min), 0.1% A (14-16 min) and 96% A (16.1-20 min). The injection volume was set at 10 µL. The parameters of both +ESI and -ESI were 2,500 V capillary voltage, 500 V end plate offset, 3 bar nebulizer, 8 L/min drying gas, 200 °C dry temperature, 200 °C probe gas temperature and 4 L/min probe gas. Instrument was operated in broadband collision-induced dissociation (bbCID), at 3 scans per second. The MS method consisted of 2 functions, the first (low energy, LE) with a collision energy of 6 eV (+ESI) and 8 eV (-ESI), and the second one using a ramp of 24-36 eV for both +ESI and -ESI. Scans were performed in the range *m/z* (Da): 70–1000. Internal calibration was done in each sample after acquiring by using sodium formate measured in the first 30 seconds of each sample.

SI-3. Method validation and quantitative analysis

Method validation parameters

A semen pool (n=5) was done for validation purposes. Twenty-one aliquots of semen were analysed as described in section *2.3 Sample treatment*. Nine of these aliquots were spiked with the analytical standards (Table S1) at the beginning of the sample treatment, at 1, 5 and 20 ng/mL (3 aliquots per concentration level). Other nine aliquots were spiked with the same analytical standards at the end of the sample treatment, just before instrumental analysis. The other 3 aliquots were not spiked with any analytical standard and were considered as procedural blanks to account for analytes already present in the semen pool. The absolute recoveries (R%) of the extraction were determined by dividing the average peak area of the aliquots spiked at the beginning of the sample treatment (n=3 per concentration level) by the average peak area of the aliquots spiked just before the instrumental analysis (n=3 per concentration level), expressed as a percentage. The calibration curves included the following concentration levels: 0, 0.05, 0.1, 0.2, 0.5, 1, 5, 10, 50, 100 ng/mL. Chemicals identified in the pooled sample without added standards were deemed residuals and were adjusted for in calculations of extraction recoveries, matrix effects, and matrix-matched calibration curves.

SI-4. Sample treatment optimization.

Sample preparation for Method 2.

Method 2 was similar to Method 1, but a preconcentration step was included. Thus, 150 µL of the pooled semen undergone to the same protocol than Method 1, but the supernatant after protein precipitation was diluted with 30 mL of Milli-Q water and passed through a SPE cartridge. The SPE cartridge was conditioned before loading the sample. The cartridge preparation and conditioning as well as the elution and reconstitution conditions were also described elsewhere (Gago-Ferrero et al., 2020). Finally, reconstitution in Method 2 was done to a final volume equivalent to the initial sample volume (150 µL), therefore, avoiding the dilution.

REFERENCE. Gago-Ferrero et al., 2020. Wide-scope target screening of >2000 emerging contaminants in wastewater samples with UPLC-Q-ToF-HRMS/MS and smart evaluation of its performance through the validation of 195 selected representative analytes. Journal of Hazardous Materials, vol. 387, 121712. DOI: 10.1016/j.jhazmat.2019.121712

SI-5. Method validation.

Table S.3. *Performance of the method validation*

Name	Ionization mode	LOQ (ng/mL)	Recovery			Repeatability	Linearity (R^2)	Matrix effect (%)	Concentration range (ng/mL)
			1 ppb (%)	5 ppb (%)	20 ppb (%)	(%)			
**2,2'-Dihydroxy-4-methoxybenzophenone**	NEG	0.2	87 ± 8	94 ± 12	107 ± 11	10%	0.995	185%	0.2 - 100
**2-Benzothiazolesulfonic acid**	NEG	0.5	27 ± 17	52 ± 10	54 ± 13	13%	0.973	104%	0.5 - 100
**3,4-Dihydroxybenzoic Acid**	NEG	0.2	65 ± 26	78 ± 11	88 ± 13	17%	0.988	161%	0.2 - 100
**Methyl 3,4-dihydroxybenzoate**	NEG	0.1	73 ± 15	98 ± 12	96 ± 10	12%	0.991	156%	0.1 - 100
**4-Hydroxybenzophenone**	NEG	0.2	96 ± 14	120 ± 9	124 ± 13	12%	0.997	115%	0.2 - 100
**6:2 Fluorotelomer sulfonate**	NEG	0.2	83 ± 13	96 ± 7	97 ± 12	11%	0.994	201%	0.2 - 100
**Benzophenone-1**	NEG	0.2	95 ± 28	99 ± 13	107 ± 18	20%	0.991	34%	0.2 - 100
**Benzophenone-2**	NEG	0.5	100 ± 20	108 ± 10	114 ± 15	15%	0.997	63%	0.5 - 100
**Benzophenone-3**	NEG	0.2	98 ± 14	127 ± 10	130 ± 11	12%	0.991	154%	0.2 - 100
**Benzophenone-4**	NEG	0.1	72 ± 7	71 ± 13	72 ± 11	10%	0.993	88%	0.1 - 100
**Benzyl paraben**	NEG	0.2	99 ± 14	129 ± 10	130 ± 12	12%	0.991	154%	0.2 - 100
**Bisphenol AP**	NEG	0.5	75 ± 17	91 ± 10	99 ± 14	14%	0.984	254%	0.5 - 100
**Bisphenol E**	NEG	0.1	42 ± 22	71 ± 24	87 ± 12	19%	0.984	158%	0.1 - 100
**Bisphenol G**	NEG	0.5	79 ± 18	96 ± 13	101 ± 9	13%	0.99	363%	0.5 - 100
**Bisphenol M**	NEG	0.2	79 ± 18	96 ± 15	104 ± 9	14%	0.99	537%	0.2 - 100
**Bisphenol P**	NEG	0.2	79 ± 18	96 ± 14	104 ± 10	14%	0.991	545%	0.2 - 100
**Bisphenol Z**	NEG	0.2	67 ± 17	82 ± 10	92 ± 12	13%	0.981	230%	0.2 - 100
**Bisphenol A**	NEG	0.2	88 ± 12	108 ± 10	112 ± 12	11%	0.994	150%	0.1 - 100
**Bisphenol AF**	NEG	0.2	87 ± 14	105 ± 10	116 ± 14	12%	0.995	282%	0.2 - 100
**Bisphenol B**	NEG	0.2	72 ± 13	88 ± 10	98 ± 11	11%	0.991	191%	0.2 - 100

**Bisphenol F**	NEG	0.5	78 ± 29	96 ± 4	107 ± 11	15%	0.996	187%	0.5 - 100
**Bisphenol S**	NEG	0.2	92 ± 29	93 ± 11	102 ± 19	20%	0.99	69%	0.2 - 100
**Clarithromycin**	NEG	10	NA[a]	NA	107 ± 22	22%	0.994	137%	10 - 100
**Di-1,2-Chloroethylphosphonic Acid**	NEG	0.2	69 ± 25	78 ± 10	86 ± 14	16%	0.982	112%	0.2 - 100
**Ethyl 3,4-Dihydroxybenzoate**	NEG	0.5	60 ± 19	76 ± 10	88 ± 11	13%	0.991	156%	0.5 - 100
**Gemfibrozil**	NEG	0.5	76 ± 22	74 ± 13	74 ± 14	16%	0.994	189%	0.5 - 100
**Isopropyl paraben**	NEG	0.5	75 ± 17	89 ± 8	101 ± 12	12%	0.988	240%	0.5 - 100
**Mecoprop**	NEG	1	26 ± 54	55 ± 22	62 ± 18	31%	0.983	156%	1 - 100
**Methyl paraben**	NEG	0.2	88 ± 15	115 ± 12	124 ± 11	13%	0.998	165%	0.2 - 100
**Mono benzyl phthalate**	NEG	1	NA	NA	95 ± 8	8%	0.995	79%	10 - 100
**Mono isobutyl phthalate**	NEG	0.5	NA	NA	90 ± 8	8%	0.995	75%	10 - 100
**Mono methyl phthalate**	NEG	0.5	NA	31 ± 46	53 ± 23	34%	0.982	71%	5 - 100
**Mono n-butyl phthalate**	NEG	0.5	NA	NA	89 ± 10	10%	0.995	56%	10 - 100
**Mono(2-ethyl-5-hydroxyhexyl) Phthalate**	NEG	0.5	53 ± 18	65 ± 15	65 ± 20	17%	0.992	274%	0.5 - 100
**Mono(2-ethyl-5-oxohexyl)phthalate**	NEG	0.2	64 ± 22	70 ± 14	81 ± 7	14%	0.994	66%	0.2 - 100
**Mono-2-ethylhexyl phatalate**	NEG	0.05	67 ± 9	93 ± 10	93 ± 14	11%	0.997	159%	1 - 100
**Monooctyl phthalate**	NEG	0.2	56 ± 11	74 ± 10	80 ± 12	11%	0.99	202%	0.2 - 100
**O-desmethyl venlafaxine**	NEG	0.1	58 ± 33	71 ± 14	80 ± 13	20%	0.996	108%	0.1 - 100
**Methyl 3,4-dichlorophenylcarbamate (SWEP)**	NEG	0.5	69 ± 14	81 ± 9	90 ± 10	11%	0.984	262%	0.5 - 100
**2-amino-Benzothiazole**	POS	0.2	138 ± 18	130 ± 21	148 ± 17	19%	0.99	63%	0.2 - 100
**2-ethylhexyl 4-(dimethylamino)benzoate**	POS	1	46 ± 30	74 ± 39	89 ± 15	28%	0.995	188%	1 - 100
**4-Aminoantipyrine**	POS	1	NA	18 ± 20	95 ± 33	43%	0.994	132%	1 - 100
**Alachlor**	POS	5	NA	29 ± 50	39 ± 15	45%	0.975	56%	5 - 100
**Carbamazepine**	POS	0.2	149 ± 10	159 ± 17	187 ± 13	13%	0.999	91%	0.2 - 100
**Carbendazim**	POS	0.1	148 ± 19	139 ± 19	152 ± 17	18%	0.999	60%	0.1 - 100

**Chlorpyriphos**	POS	5	NA	81 ± 40	88 ± 16	30%	0.995	116%	5 - 100
**Dimethomorph**	POS	5	NA	51 ± 52	39 ± 24	55%	0.996	123%	5 - 100
**Dimethyl benzotriazole**	POS	1	150 ± 15	152 ± 22	163 ± 15	18%	0.998	81%	1 - 100
**Drometrizole**	POS	5	NA	11 ± 72	102 ± 37	54%	0.983	173%	0.1 - 100
**Enrofloxacin**	POS	5	NA	27 ± 31	40 ± 29	60%	0.993	-3	5 - 100
**Enzacamene**	POS	10	NA	NA	25 ± 21	48%	0.983	84%	50 - 100
**Flumequine**	POS	0.2	34 ± 22	75 ± 38	94 ± 7	22%	0.998	68%	0.2 - 100
**Lauryl diethanolamide**	POS	10	NA	NA	41 ± 28	50%	0.997	-3	10 - 100
**Malathion**	POS	5	NA	60 ± 43	64 ± 19	30%	0.999	44%	5 - 100
**Metalaxyl**	POS	5	NA	158 ± 15	177 ± 15	22%	1.000	99%	5 - 100
**Methiocarb**	POS	5	NA	45 ± 46	46 ± 20	35%	0.995	50%	5 - 100
**Molinate**	POS	0.5	39 ± 47	69 ± 46	64 ± 22	38%	0.996	434%	0.5 - 100
**Nalidixic acid**	POS	0.2	29 ± 46	57 ± 35	63 ± 19	33%	0.983	395%	0.2 - 100
**N-desmethyl venlafaxine**	POS	0.2	75 ± 22	107 ± 26	118 ± 21	23%	0.99	158%	0.2 - 100
**Nicotine**	POS	0.2	70 ± 40	81 ± 34	79 ± 20	31%	0.979	179%	0.2 - 100
**Oxadiazon**	POS	0.5	54 ± 18	68 ± 40	80 ± 18	25%	0.99	44%	0.5 - 100
**Oxolinic acid**	POS	1	74 ± 29	91 ± 26	103 ± 21	25%	0.995	198%	1 - 100
**Phenazone**	POS	5	NA	85 ± 36	94 ± 24	55%	0.994	155%	5 - 100
**Propanil**	POS	1	31 ± 40	38 ± 45	42 ± 24	36%	0.997	81%	1 - 100
**Sulfadiazine**	POS	1	25 ± 101	33 ± 60	28 ± 19	60%	0.985	138%	1 - 100
**Sulfamerazine**	POS	0.2	80 ± 11	79 ± 25	90 ± 14	17%	0.984	132%	0.2 - 100
**Sulfamethoxazole**	POS	0.05	88 ± 20	100 ± 25	115 ± 16	20%	0.993	47%	0.05 - 100
**Tris(2-chloroethyl) phosphate (TCEP)**	POS	5	NA	52 ± 46	56 ± 16	29%	0.999	22%	5 - 100
**Tris(2-chloropropyl) phosphate**	POS	5	NA	NA	56 ± 19	42%	0.988	45%	50 - 100
**Terbumeton**	POS	1	138 ± 11	158 ± 17	183 ± 11	13%	0.999	107%	1 - 100
**Tetradecylamine**	POS	10	NA	NA	7 ± 29	50%	0.974	-3	10 - 100
**Tonalide**	POS	5	NA	102 ± 43	95 ± 17	35%	0.997	187%	5 - 100

**Tri-o-tolyl phosphate**	POS	2	NA	79 ± 41	91 ± 15	29%	0.999	-3	5 - 100
**Tryptamine**	POS	10	NA	117 ± 28	115 ± 15	28%	0.996	49%	10 - 100
**Tryptoline**	POS	1	47 ± 40	101 ± 28	106 ± 17	28%	0.997	26%	1 - 100
**Umbelliferone**	POS	10	NA	NA	134 ± 35	31%	0.998	32%	10 - 100
**Venlafaxine**	POS	0.2	94 ± 27	111 ± 26	122 ± 21	24%	0.986	178%	0.2 - 100
**Zoxamide**	POS	5	NA	66 ± 46	62 ± 17	34%	0.984	38%	5 - 100
**Diuron**	POS / NEG	1	179 ± 16	169 ± 16	191 ± 13	15%	0.998	82%	1 - 100
**Methyl-benzotriazole**	POS / NEG	1	30 ± 37	78 ± 12	93 ± 13	21%	0.991	134%	1 - 100
**Monocyclohexyl phthalate**	POS / NEG	0.1	53 ± 9	70 ± 12	73 ± 15	12%	0.996	174%	0.1 - 100
**Mono(5-carboxy-2-ethylpentyl) Phthalate**	POS / NEG	0.1	29 ± 21	36 ± 21	36 ± 20	20%	0.99	375%	0.1 - 100
**Monopentyl Phthalate**	POS / NEG	0.5	56 ± 9	64 ± 11	69 ± 14	11%	0.992	165%	0.5 - 100
**N-acetyl sulfadiazine**	POS / NEG	0.5	69 ± 35	62 ± 17	58 ± 12	21%	0.983	41%	0.5 - 100
**N-acetyl sulfamethazine**	POS / NEG	0.2	59 ± 21	67 ± 27	70 ± 12	20%	0.991	155%	0.2 - 100
**N-acetyl sulfapyridine**	POS / NEG	0.5	77 ± 23	83 ± 10	91 ± 26	20%	0.992	100%	0.5 - 100
**Sulfadimethoxine**	POS / NEG	1	70 ± 38	92 ± 8	98 ± 14	20%	0.997	30%	1 - 100
**Sulfamethoxypyridazine**	POS / NEG	0.2	94 ± 17	103 ± 23	123 ± 16	18%	0.994	67%	0.2 - 100
**Sulfaquinoxaline**	POS / NEG	1	106 ± 47	100 ± 9	98 ± 15	24%	0.996	22%	1 - 100
**Sulfathiazole**	POS / NEG	0.5	50 ± 32	76 ± 11	88 ± 15	19%	0.997	10%	0.5 - 100
**Triclocarban**	POS / NEG	5	NA	70 ± 41	83 ± 15	23%	0.998	70%	5 - 100

[a]Values are not available (NA) as the LOQ was not enough to calculate the recoveries.

SI-6. Wide-scope screening of polar and semi-polar organic chemicals in samples of human seminal plasma.

Table S.4. *Distribution of analyte concentrations in each sample.*

Name	InChiKey[a]	LOQ (ng/mL)	Sample code									
			**1**	**2**	**3**	**4**	**5**	**6**	**7**	**8**	**9**	**10**
**2-Mercapto-benzothiazole (MBT)**	YXIWHUQXZSMYRE-UHFFFAOYSA-N	0.1	0.8	nd	nd	nd	<LOQ	nd	nd	nd	nd	nd
**Bisphenol A**	IISBACLAFKSPIT-UHFFFAOYSA-N	0.2	nd	nd	nd	nd	nd	nd	nd	nd	nd	<LOQ
**Bisphenol S**	VPWNQTHUCYMVMZ-UHFFFAOYSA-N	0.2	nd	0.25	nd	nd	nd	nd	nd	nd	nd	nd
**C12-LAS**	YNJLGQRDQAQAHR-UHFFFAOYSA-N	30[b]	68	47	180	95	49	60	46	53	51	159
**C14-LAS**	OJUCYEORNQUZQE-UHFFFAOYSA-N	20[b]	40	24	77	63	24	28	26	28	27	74
**Caffeine**	RYYVLZVUVIJVGH-UHFFFAOYSA-N	5	1,964	93	1,887	3,660	22	1,074	1,365	1,489	125	3,112
**DEET**	MMOXZBCLCQITDF-UHFFFAOYSA-N	10	35	27	19	52	nd	nd	24	47	33	30
**Dioctylsulfosuccinate**	IGHFHMYYZNNSKX-UHFFFAOYSA-N	0.05	0.2	0.1	1.3	0.3	0.2	0.1	2.9	0.5	0.3	2.0
**Diphenylphosphate**	ASMQGLCHMVWBQR-UHFFFAOYSA-N	0.5[b]	9.5	2.1	1.6	<LOQ	12	11	9.4	2.6	0.9	8.3
**Mono 2-ethylhexyl phthalate**	DJDSLBVSSOQSLW-UHFFFAOYSA-M	0.05[b]	nd	0.06	<LOQ	<LOQ	nd	<LOQ	<LOQ	nd	<LOQ	nd
**Mono butyl + isobutyl phthalate**	YZBOVSFWWNVKRJ-UHFFFAOYSA-N + RZJSUWQGFCHNFS-UHFFFAOYSA-N	0.5	nd	<LOQ	nd	nd	0.7	2.1	1.7	nd	3.2	nd
**N,N-Dimethyldodecylamine**	YWFWDNVOPHGWMX-UHFFFAOYSA-N	5[b]	7.0	19	18	110	7.7	11	16	38	30	41
**PFBS**	JGTNAGYHADQMCM-UHFFFAOYSA-N	0.1[b]	nd	nd	nd	nd	nd	nd	nd	nd	nd	0.3
**PFOA**	SNGREZUHAYWORS-UHFFFAOYSA-N	0.005[b]	0.02	0.01	0.02	0.04	0.02	0.01	0.02	0.01	0.02	0.02
**PFOS**	YFSUTJLHUFNCNZ-UHFFFAOYSA-N	0.05[b]	0.5	0.3	0.3	1.4	0.7	0.1	0.4	0.3	0.5	0.9
**Theobromine**	YAPQBXQYLJRXSA-UHFFFAOYSA-N	5	201	85	501	378	187	87	464	168	190	900
**Theophylline**	ZFXYFBGIUFBOJW-UHFFFAOYSA-N	20	585	38	376	819	nd	144	417	228	85	1,200
**Tributylamine**	IMFACGCPASFAPR-UHFFFAOYSA-N	1	5.7	2.2	5.2	2.4	nd	nd	2.9	2.4	2.8	4.6
**Tri-o-tolyl phosphate**	YSMRWXYRXBRSND-UHFFFAOYSA-N	2	nd	4.9	<LOQ	nd	<LOQ	nd	nd	nd	<LOQ	nd
**Triphenyl phosphate**	XZZNDPSIHUTMOC-UHFFFAOYSA-N	2	3.1	3.2	4.0	3.3	2.9	3.0	2.7	2.5	3.6	3.2

[a]*Computed by InChI 1.0.6 (PubChem release 2021.05.07).*[b]*Estimated as the average concentration of the blanks plus three times the standard deviation. Abbreviations: non-detected (nd), under the limit of quantification (<LOQ), perfluorobutane sulfonic acid (PFBS), perfluorooctanoic acid (PFOA), perfluorooctane sulfonate (PFOS), diethyltoluamide (DEET), dodecyl sulfate* (C12-*LAS) and tetradecyl sulfate* (C14-*LAS)*

Figure S.1. Chromatograms for all identified chemicals.

Clarification: We selected the chromatograms of the samples that had the lowest concentration, ensuring that the peak was the one closest to the detectability limit. Five chemicals did not exhibit fragmentation due to the unspecific collision energy utilized in non-targeted experiments. Nonetheless, given that they were injected into our instrument and displayed matching retention time (RT) and accurate mass, they are reported as detected.

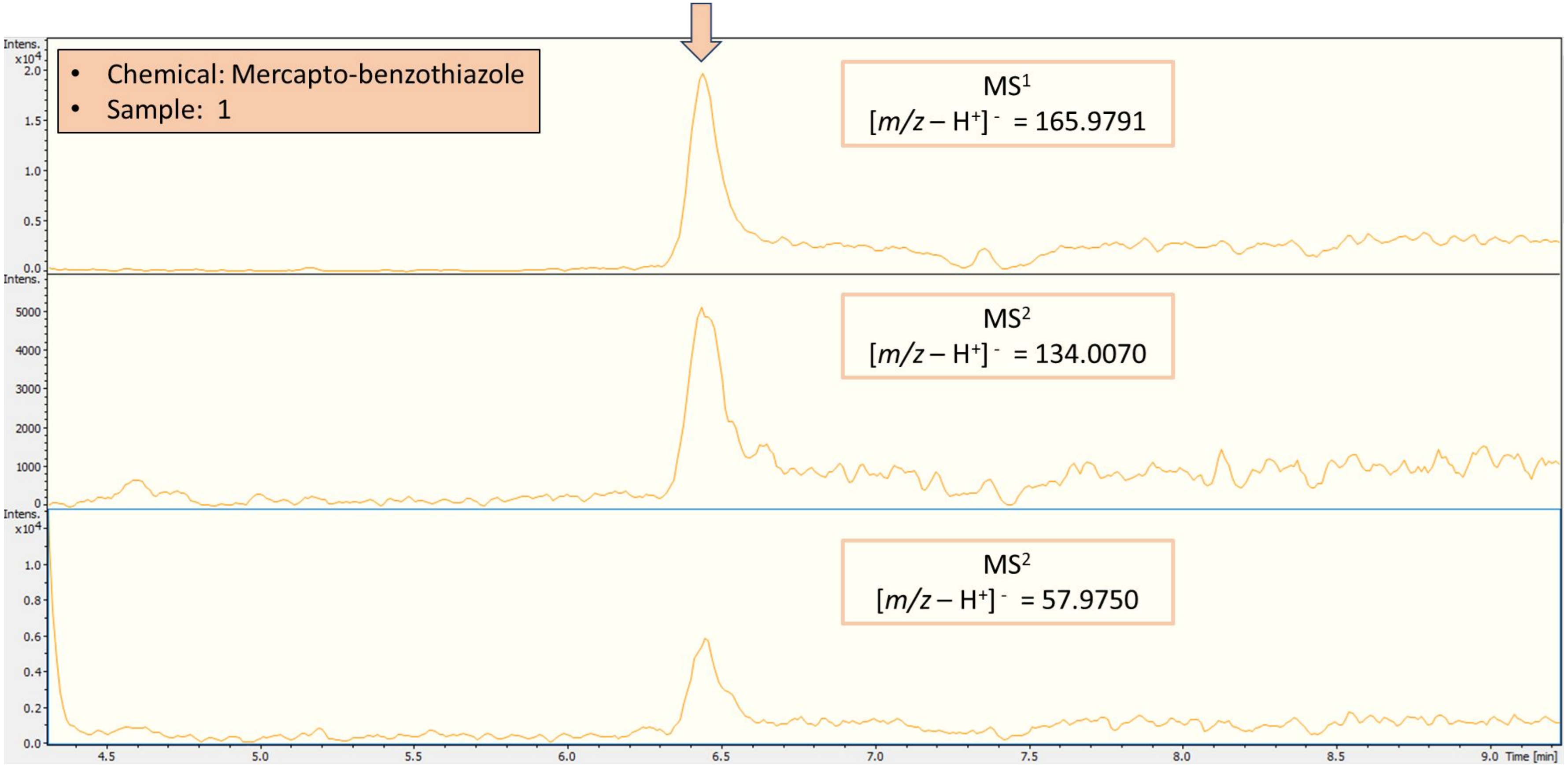

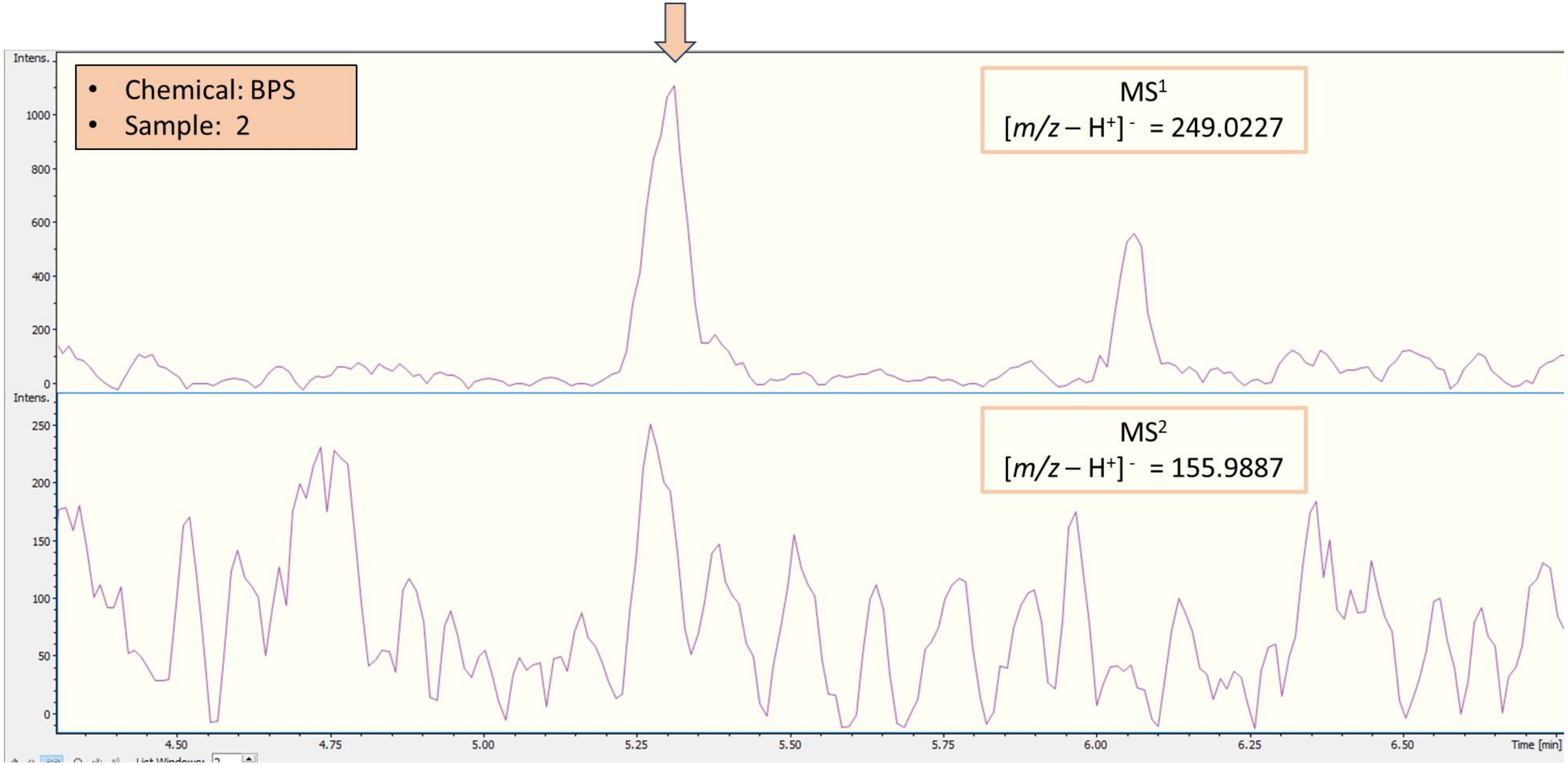

Chemical: BPS
Sample: 2
MS1
[m/z – H+]- = 249.0227
MS2
[m/z – H+]- = 155.9887
Intens.
Time [min]

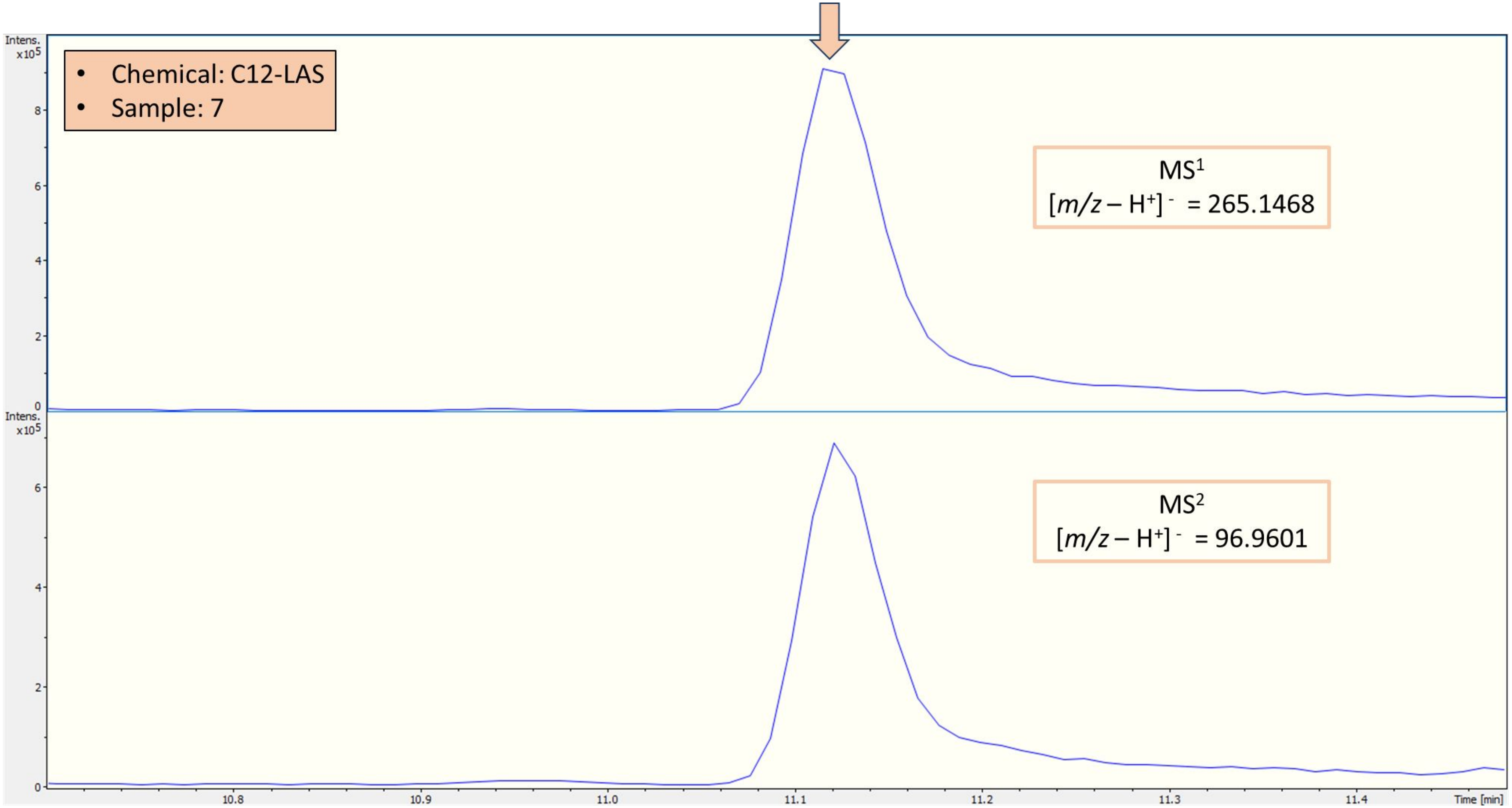
Intens. x10^5
Chemical: C12-LAS
Sample: 7
MS1
[m/z – H+]- = 265.1468
MS2
[m/z – H+]- = 96.9601
10.8
10.9
11.0
11.1
11.2
11.3
11.4
Time [min]

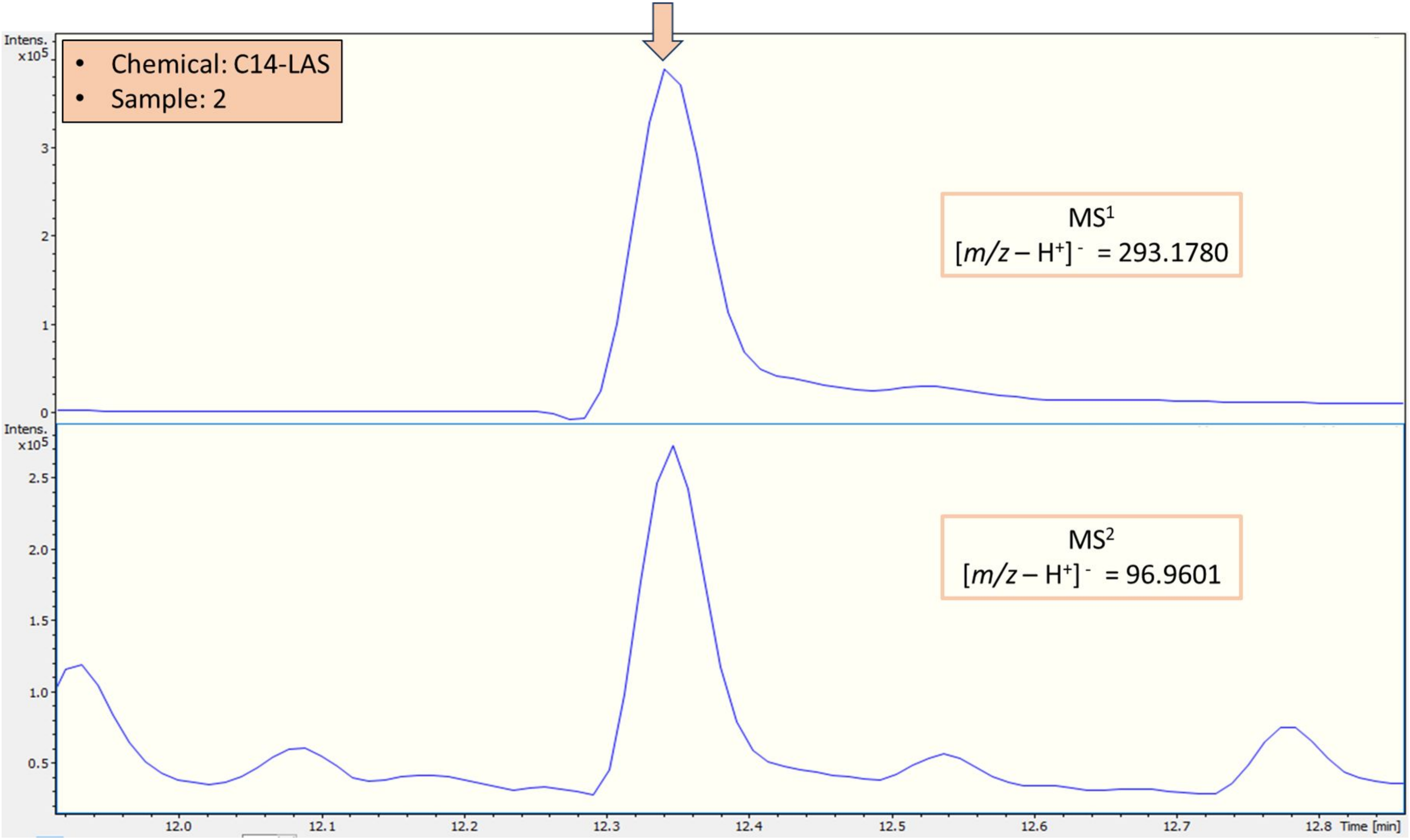

Intens.
x10^5
Chemical: C14-LAS
Sample: 2
MS1
[m/z – H+]- = 293.1780
MS2
[m/z – H+]- = 96.9601
Intens.
x10^5
12.0
12.1
12.2
12.3
12.4
12.5
12.6
12.7
12.8
Time [min]

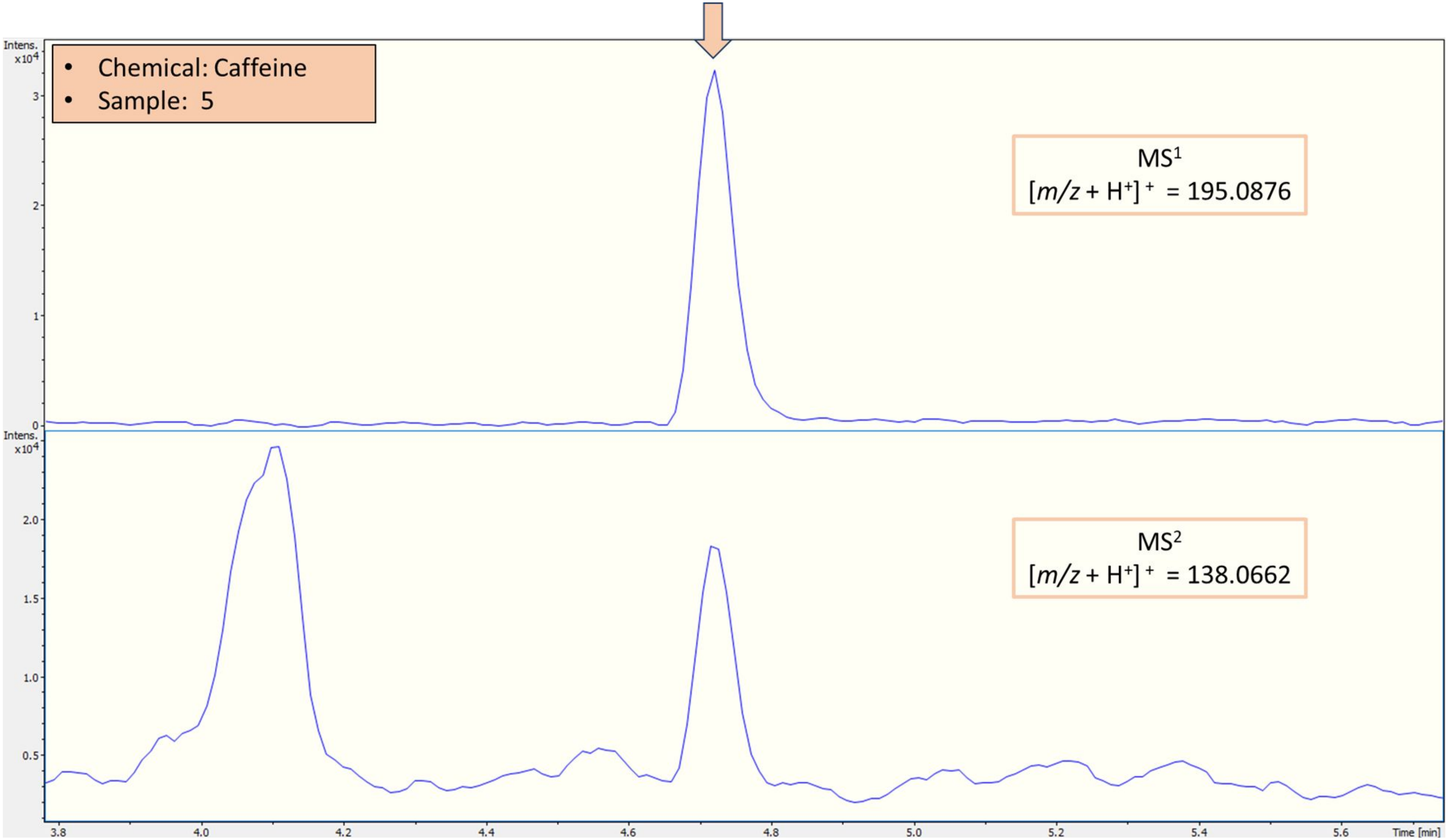

Chemical: Caffeine
Sample: 5
MS1
[m/z + H+]+ = 195.0876
MS2
[m/z + H+]+ = 138.0662
Intens. x10^4
Time [min]

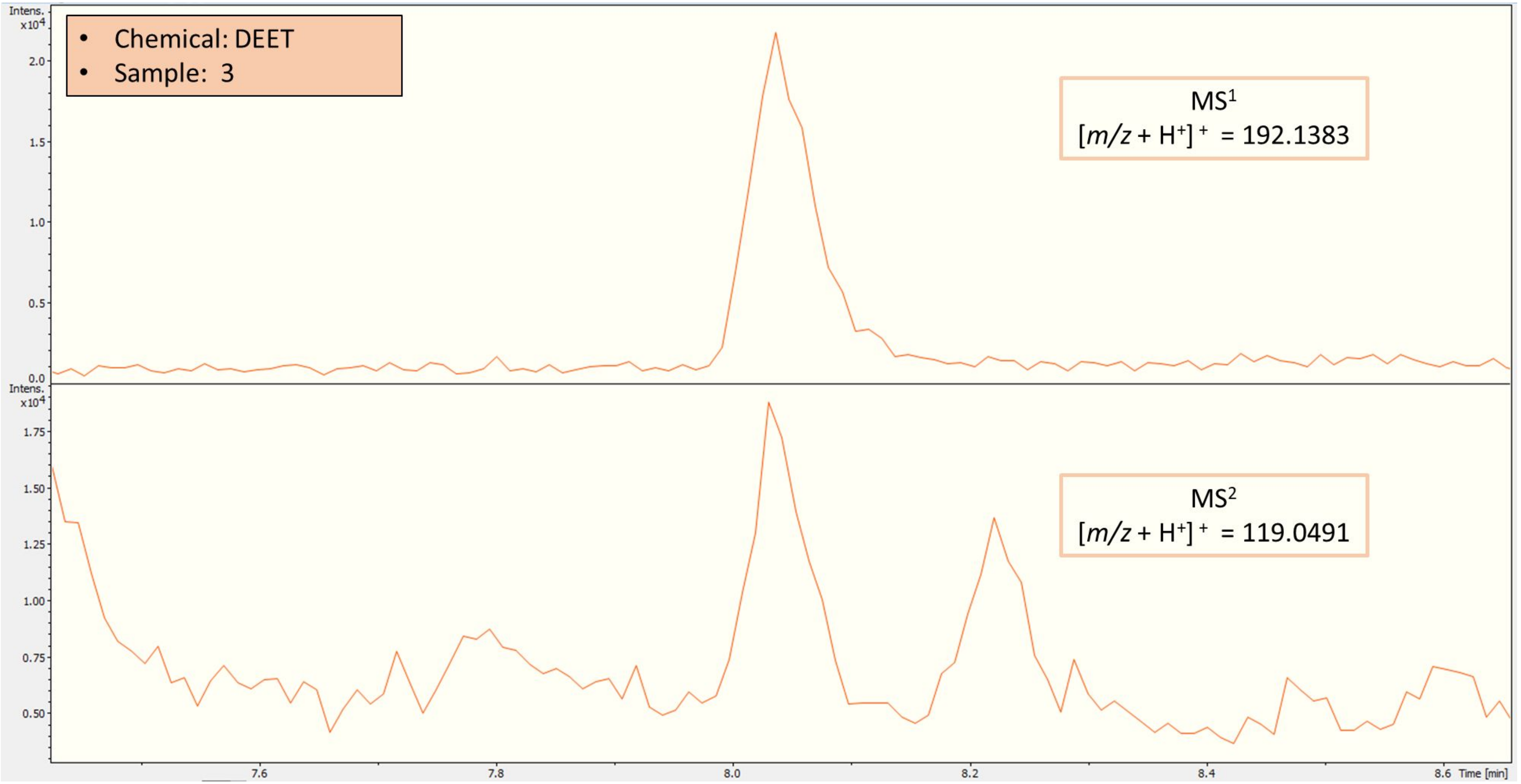

Chemical: DEET
Sample: 3
MS1
[m/z + H+]+ = 192.1383
MS2
[m/z + H+]+ = 119.0491
Intens. x10^4
2.0
1.5
1.0
0.5
0.0
Intens. x10^4
1.75
1.50
1.25
1.00
0.75
0.50
7.6
7.8
8.0
8.2
8.4
8.6
Time [min]

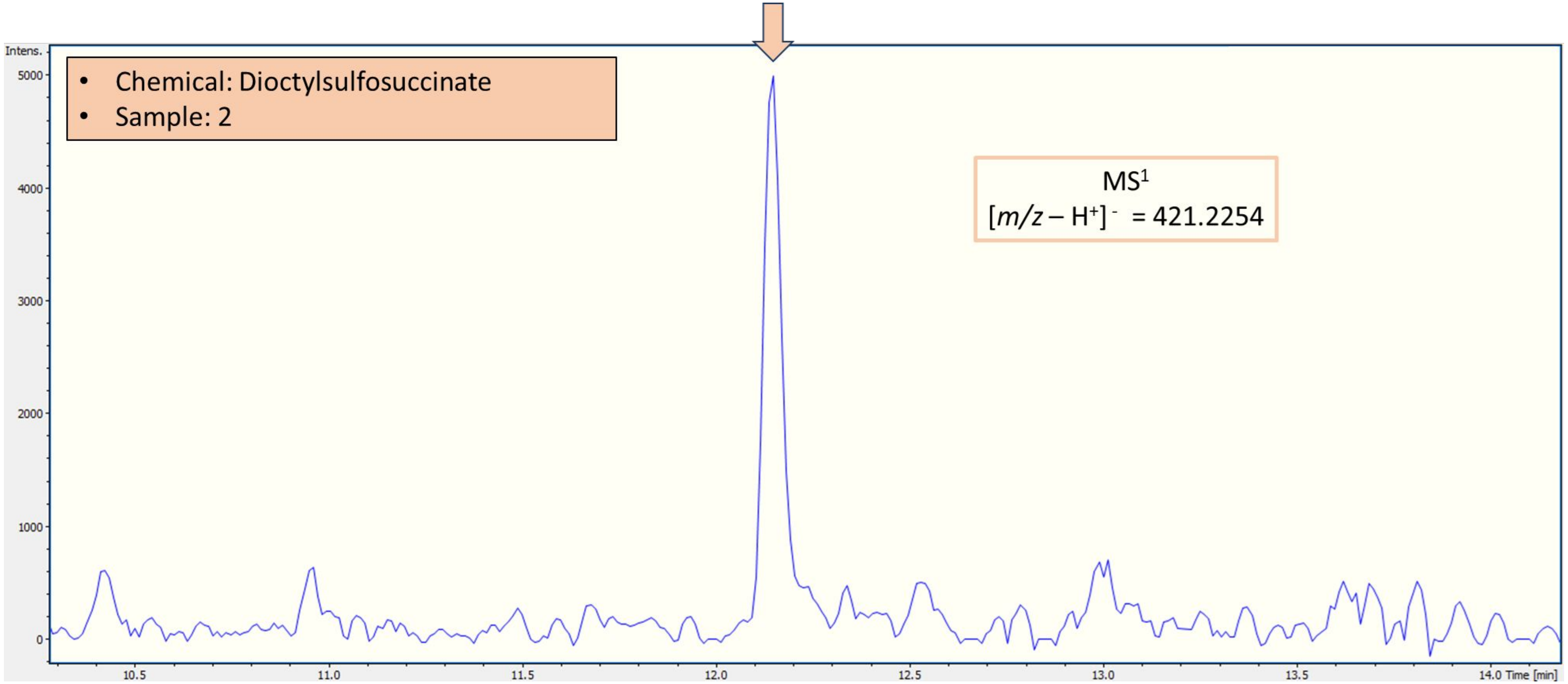

Chemical: Dioctylsulfosuccinate
Sample: 2
MS1
[m/z – H+]- = 421.2254
Intens.
5000
4000
3000
2000
1000
0
10.5
11.0
11.5
12.0
12.5
13.0
13.5
14.0 Time [min]

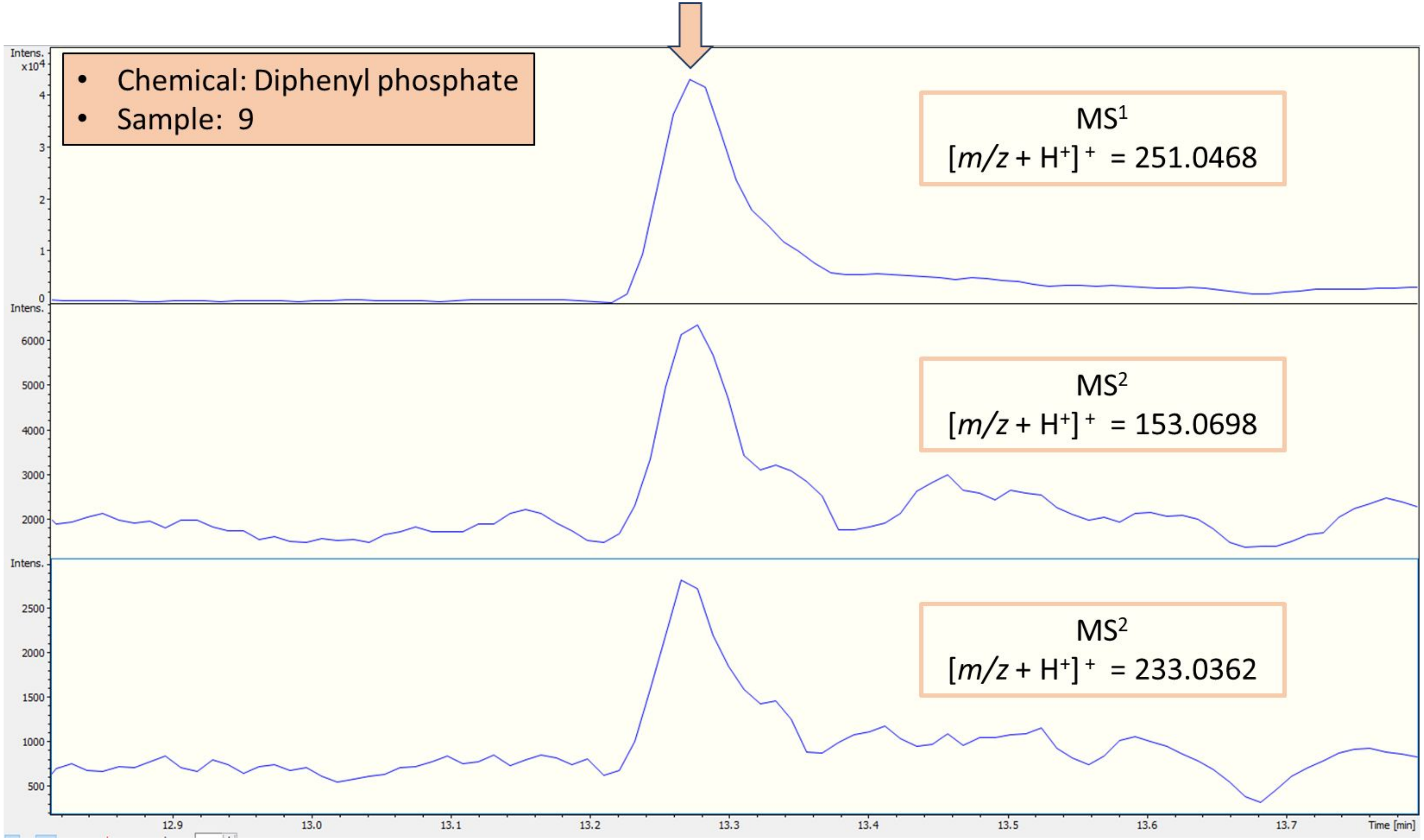

Chemical: Diphenyl phosphate
Sample: 9
MS1
[m/z + H+]+ = 251.0468
MS2
[m/z + H+]+ = 153.0698
MS2
[m/z + H+]+ = 233.0362
Intens.
x10^4
Time [min]

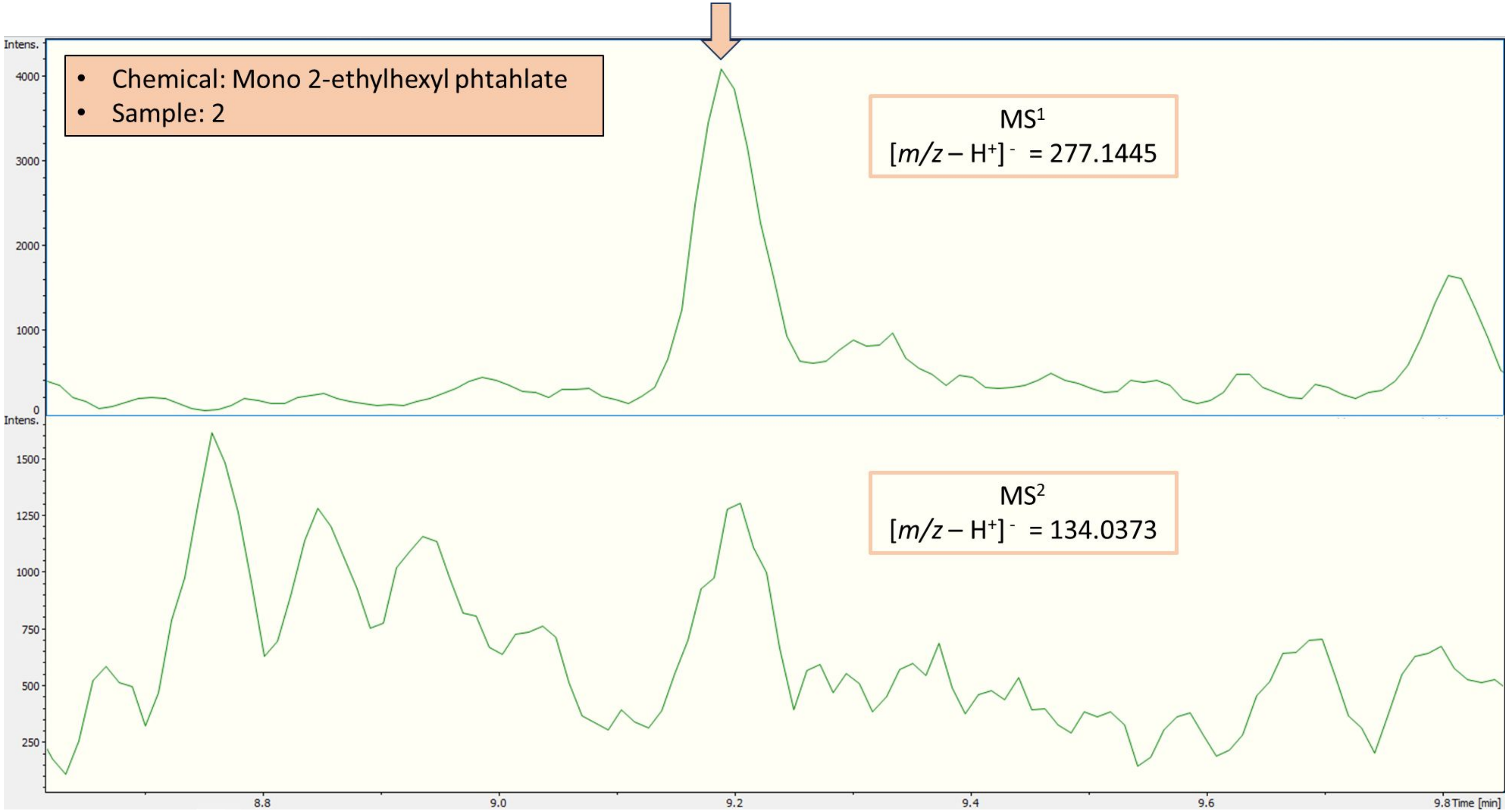

Chemical: Mono 2-ethylhexyl phtahlate
Sample: 2
MS1
[m/z – H+]- = 277.1445
MS2
[m/z – H+]- = 134.0373
Intens.
4000
3000
2000
1000
0
Intens.
1500
1250
1000
750
500
250
8.8
9.0
9.2
9.4
9.6
9.8 Time [min]

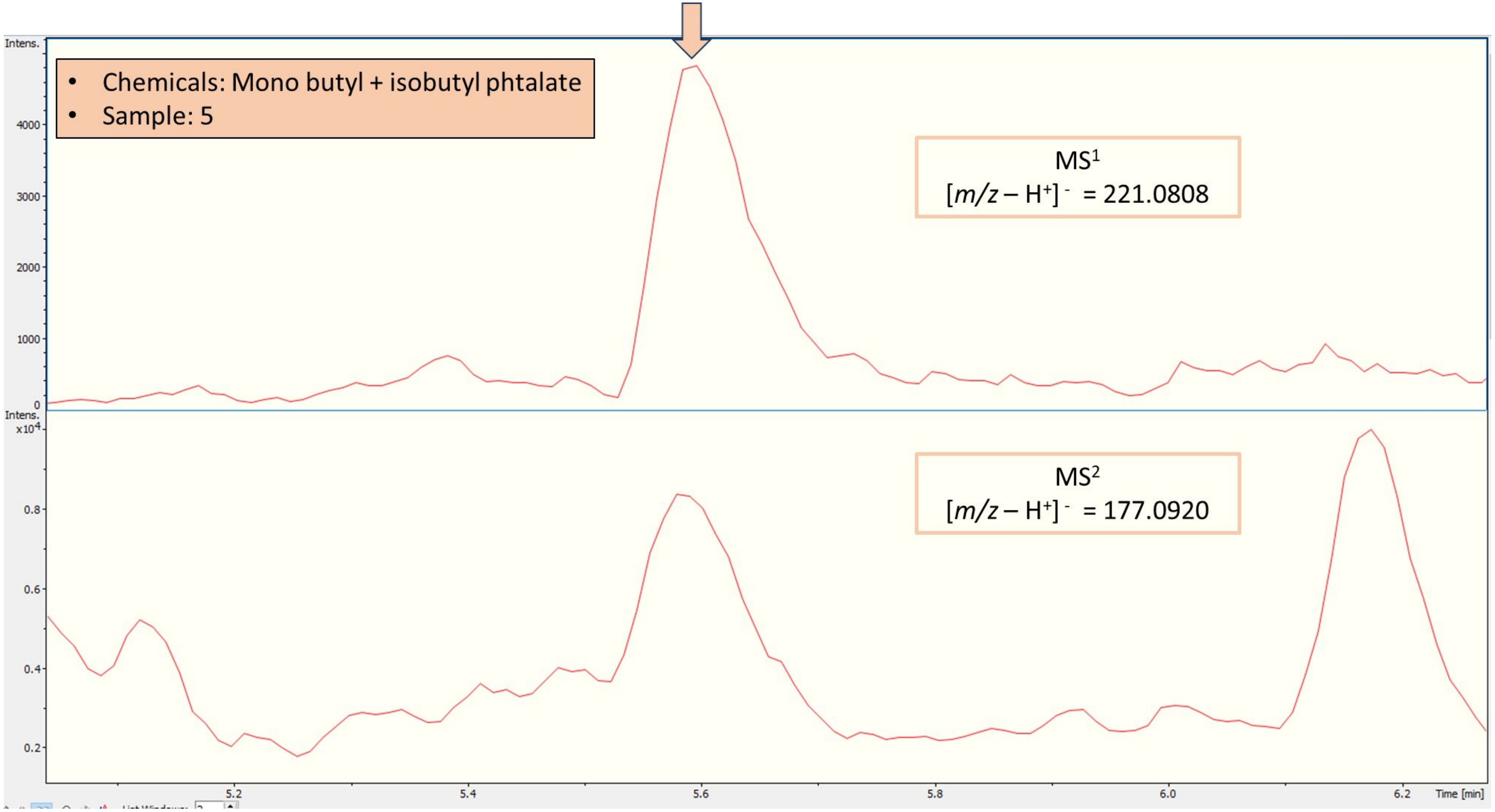
Chemicals: Mono butyl + isobutyl phtalate
Sample: 5
MS1
[m/z – H+]- = 221.0808
MS2
[m/z – H+]- = 177.0920
Intens.
Intens.
x10^4
Time [min]

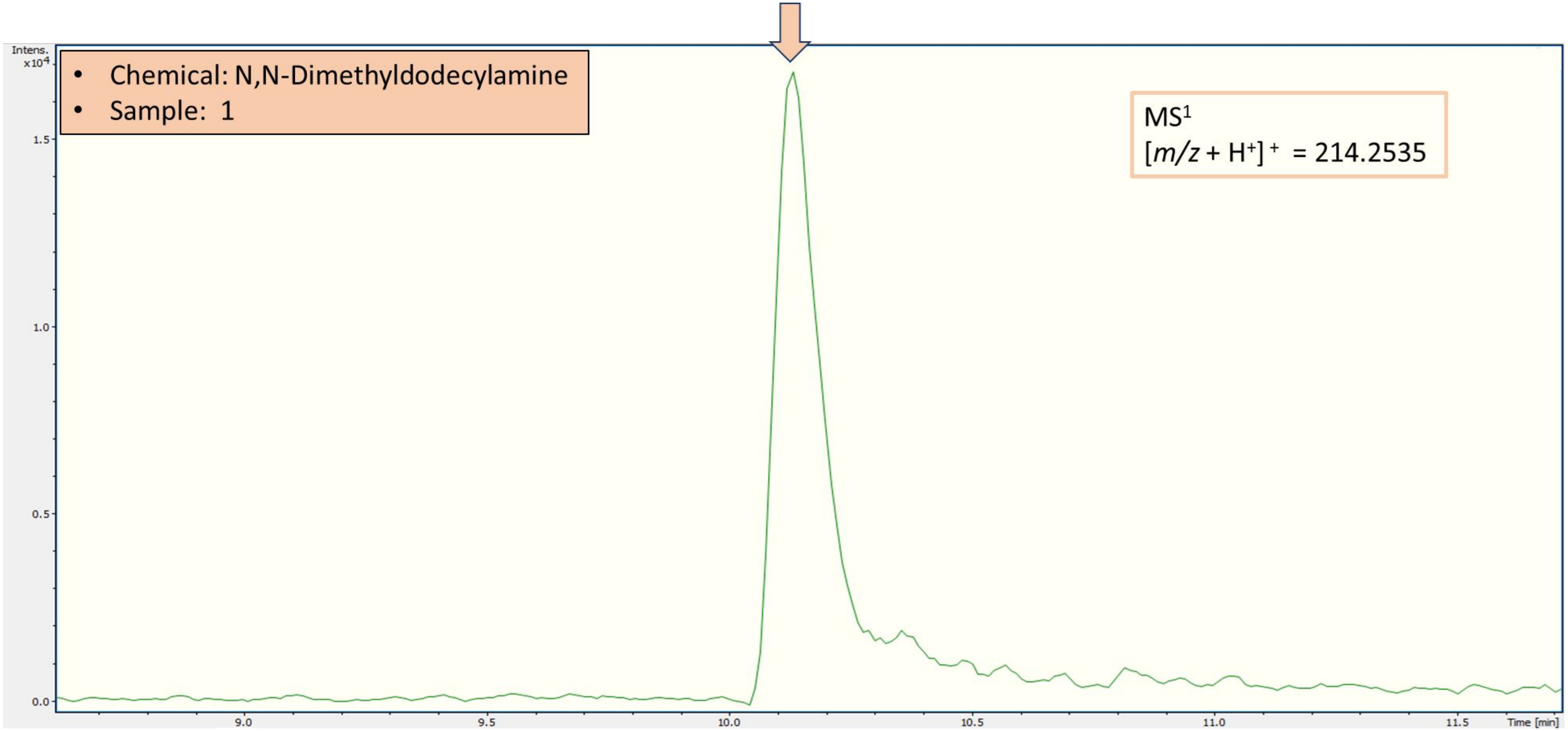

Intens.
x10^4
Chemical: N,N-Dimethyldodecylamine
Sample: 1
MS1
[m/z + H+]+ = 214.2535
1.5
1.0
0.5
0.0
9.0
9.5
10.0
10.5
11.0
11.5
Time [min]

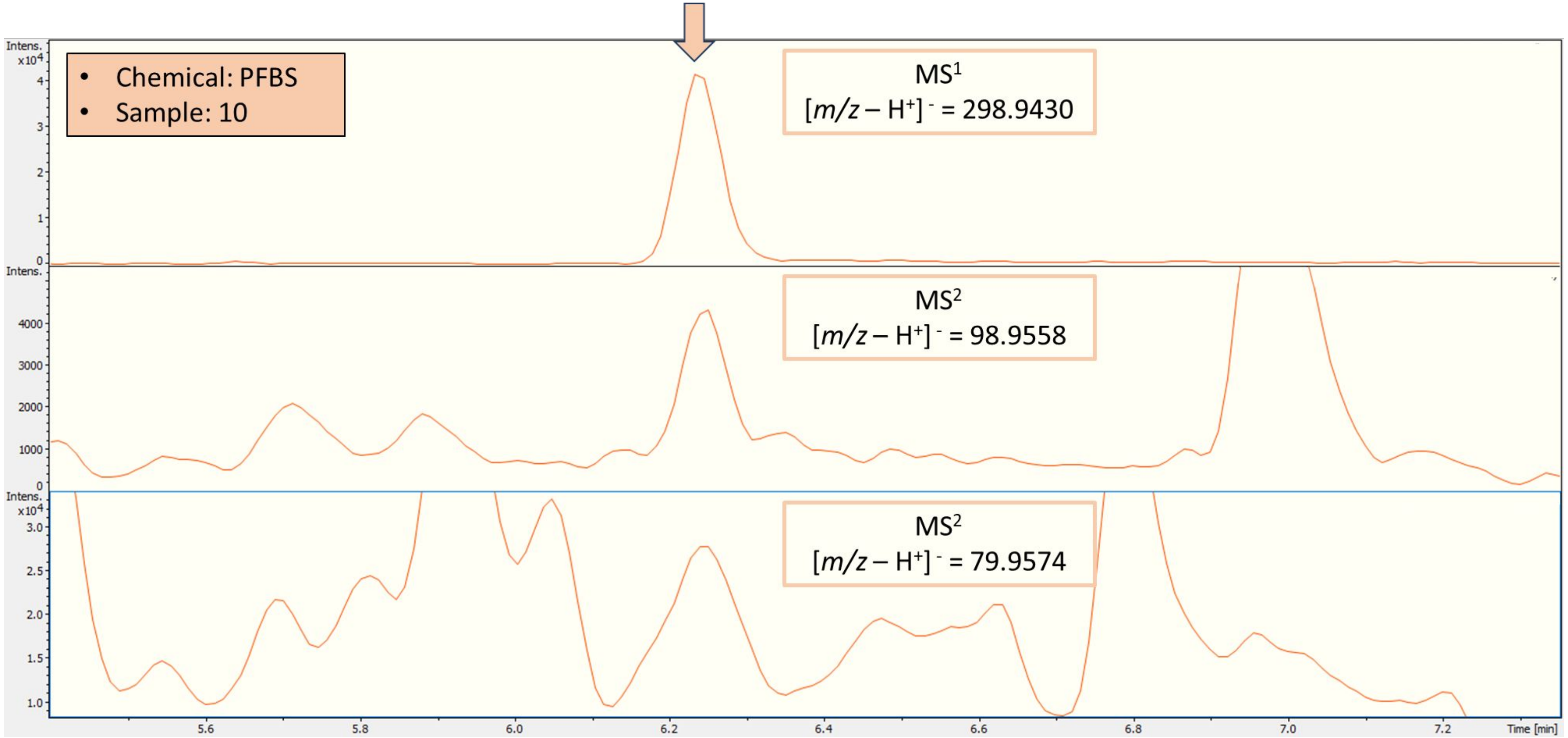

Chemical: PFBS
Sample: 10
MS1
[m/z – H+] - = 298.9430
MS2
[m/z – H+] - = 98.9558
MS2
[m/z – H+] - = 79.9574
Intens.
x10^4
Time [min]

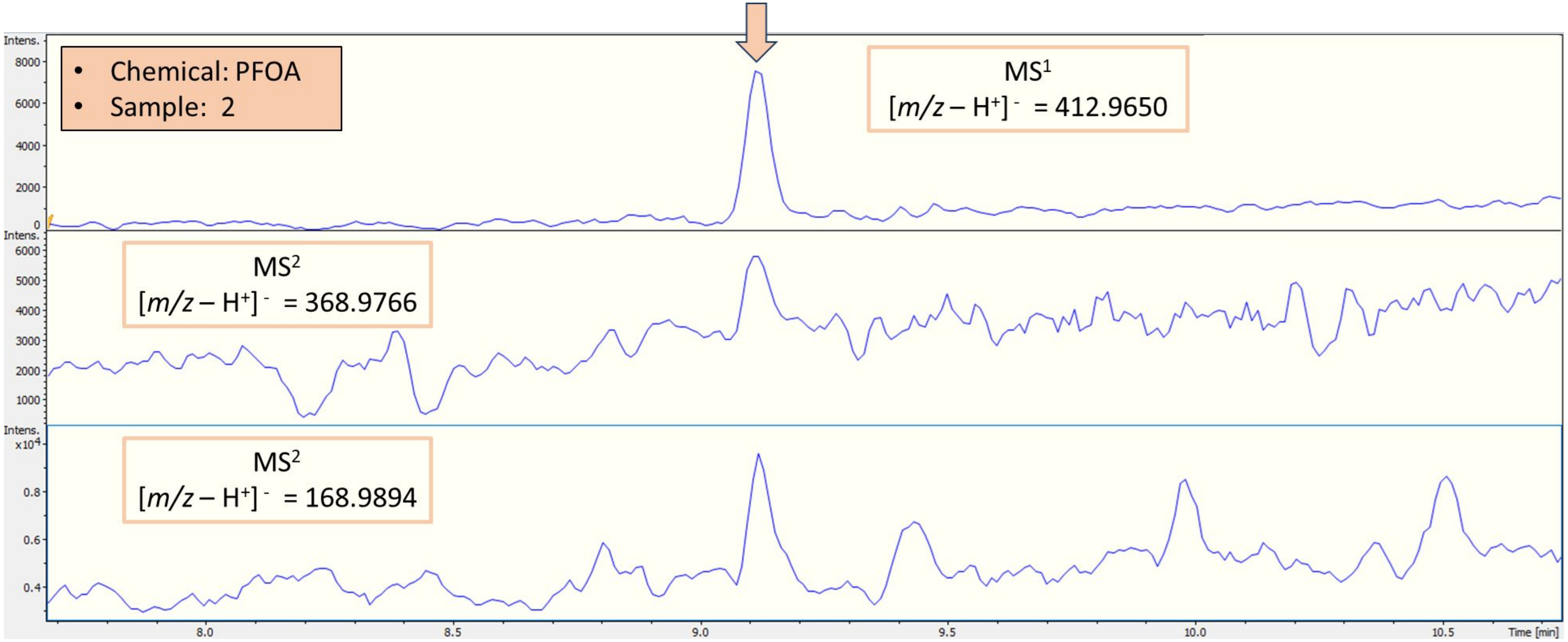

Chemical: PFOA
Sample: 2
MS1
[m/z – H+] - = 412.9650
MS2
[m/z – H+] - = 368.9766
MS2
[m/z – H+] - = 168.9894
Intens.
x10^4
Time [min]

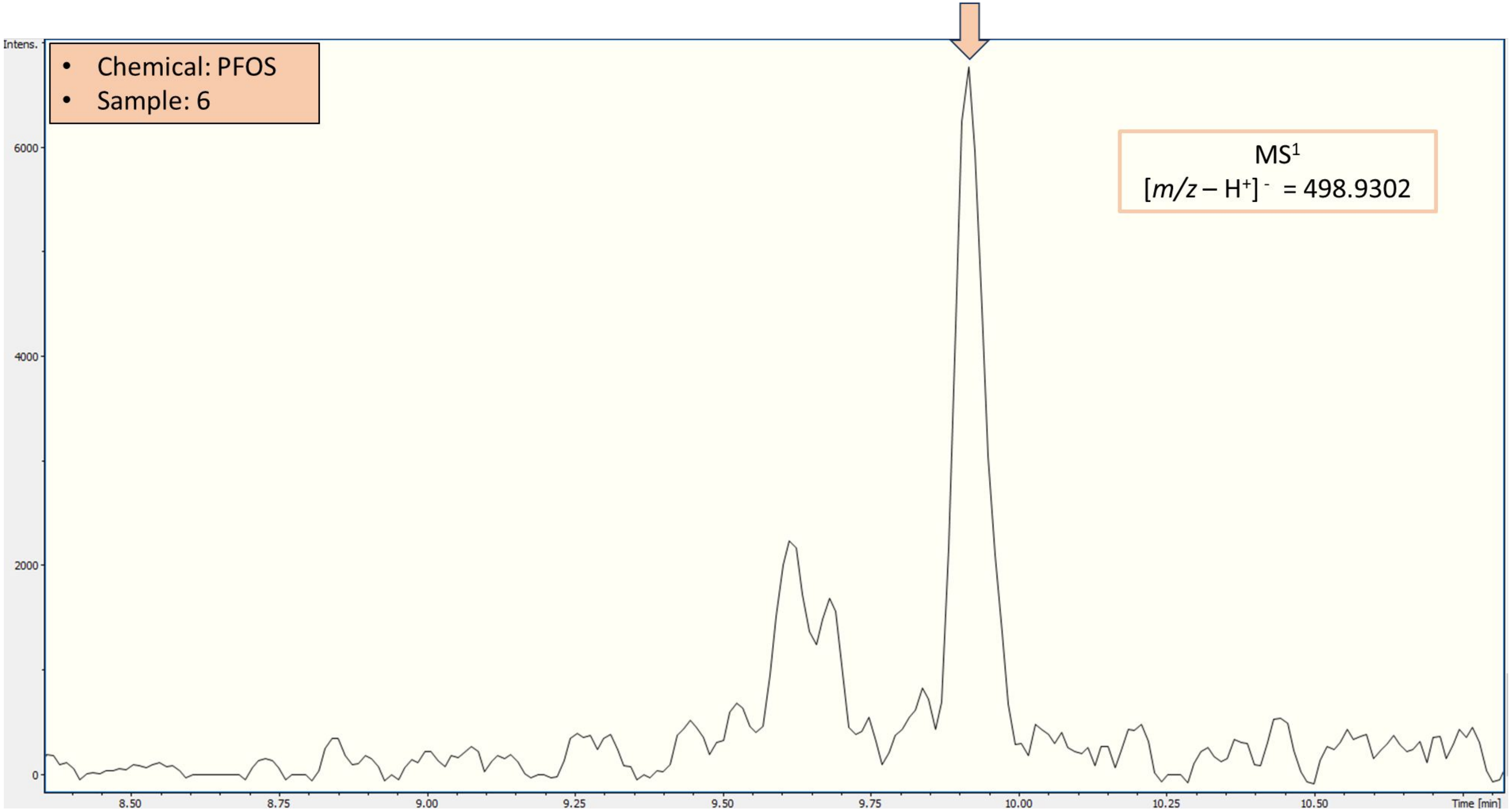
Chemical: PFOS
Sample: 6
MS1
[m/z – H+]- = 498.9302
Intens.
6000
4000
2000
0
8.50
8.75
9.00
9.25
9.50
9.75
10.00
10.25
10.50
Time [min]

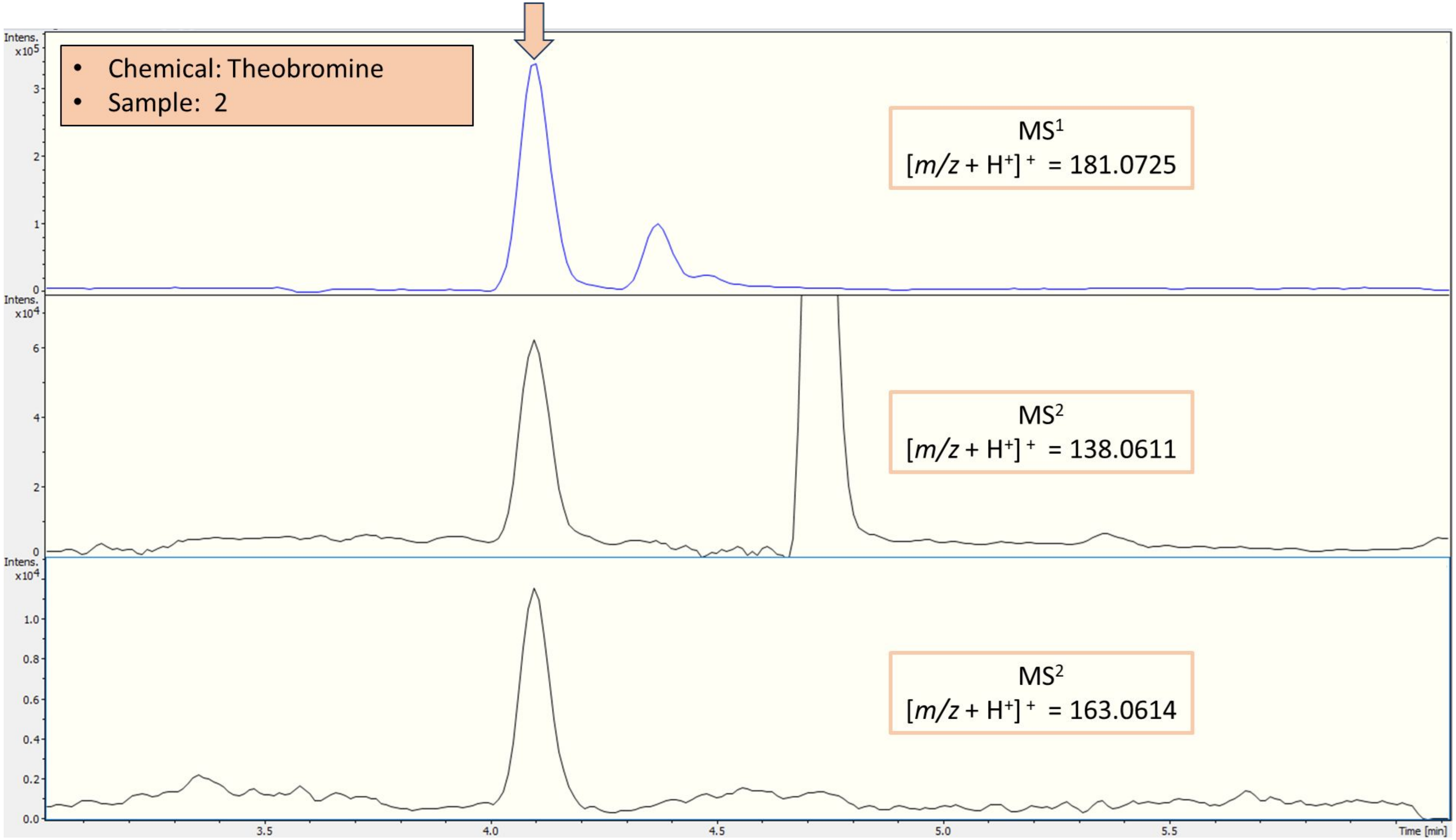
Chemical: Theobromine
Sample: 2
MS1
[m/z + H+]+ = 181.0725
MS2
[m/z + H+]+ = 138.0611
MS2
[m/z + H+]+ = 163.0614
Intens. x10^5
Intens. x10^4
Intens. x10^4
Time [min]
3.5
4.0
4.5
5.0
5.5

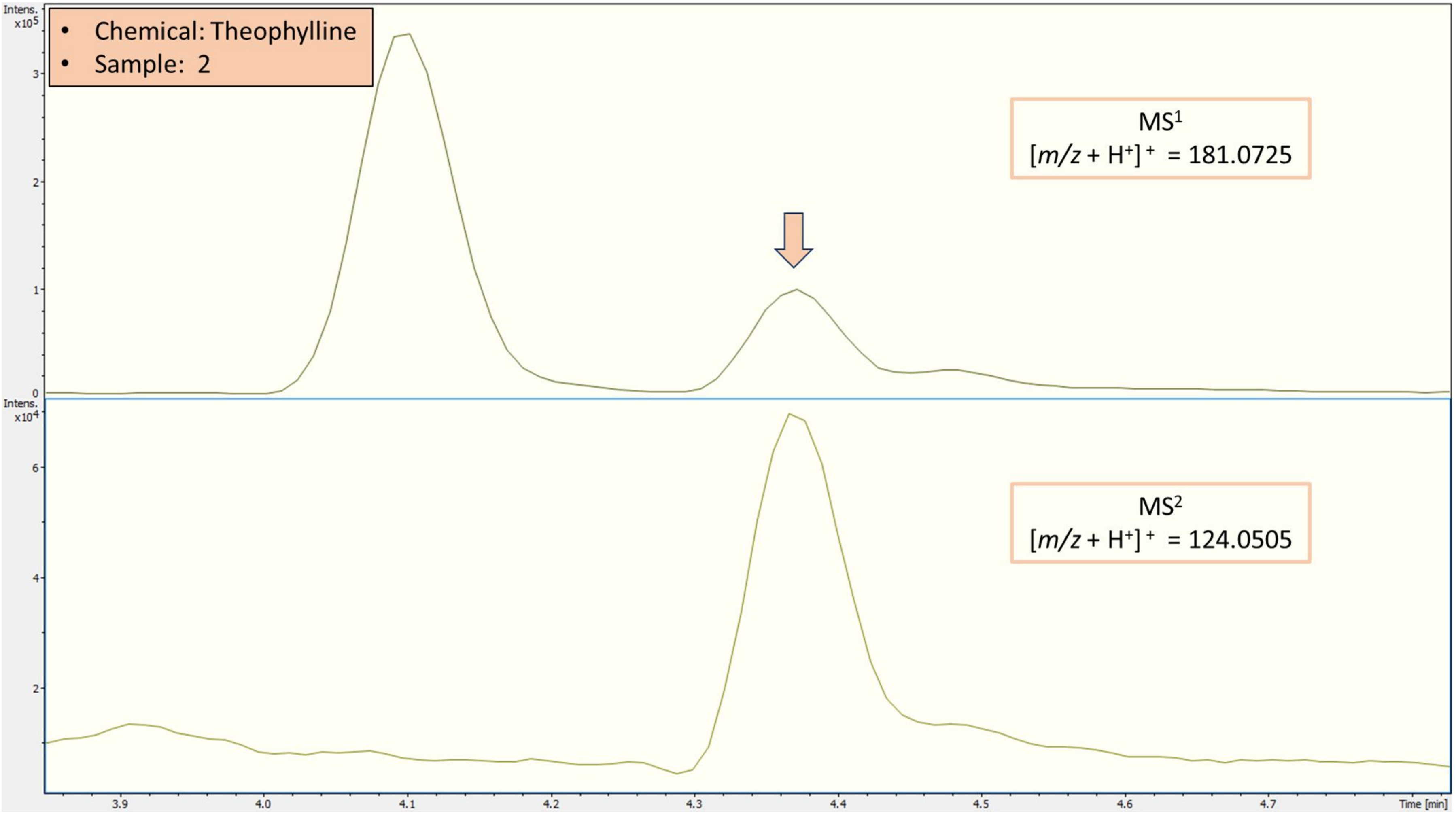
Chemical: Theophylline
Sample: 2
MS^1
[m/z + H$^+$]$^+$ = 181.0725
MS^2
[m/z + H$^+$]$^+$ = 124.0505
Intens. x10^5
Intens. x10^4
Time [min]

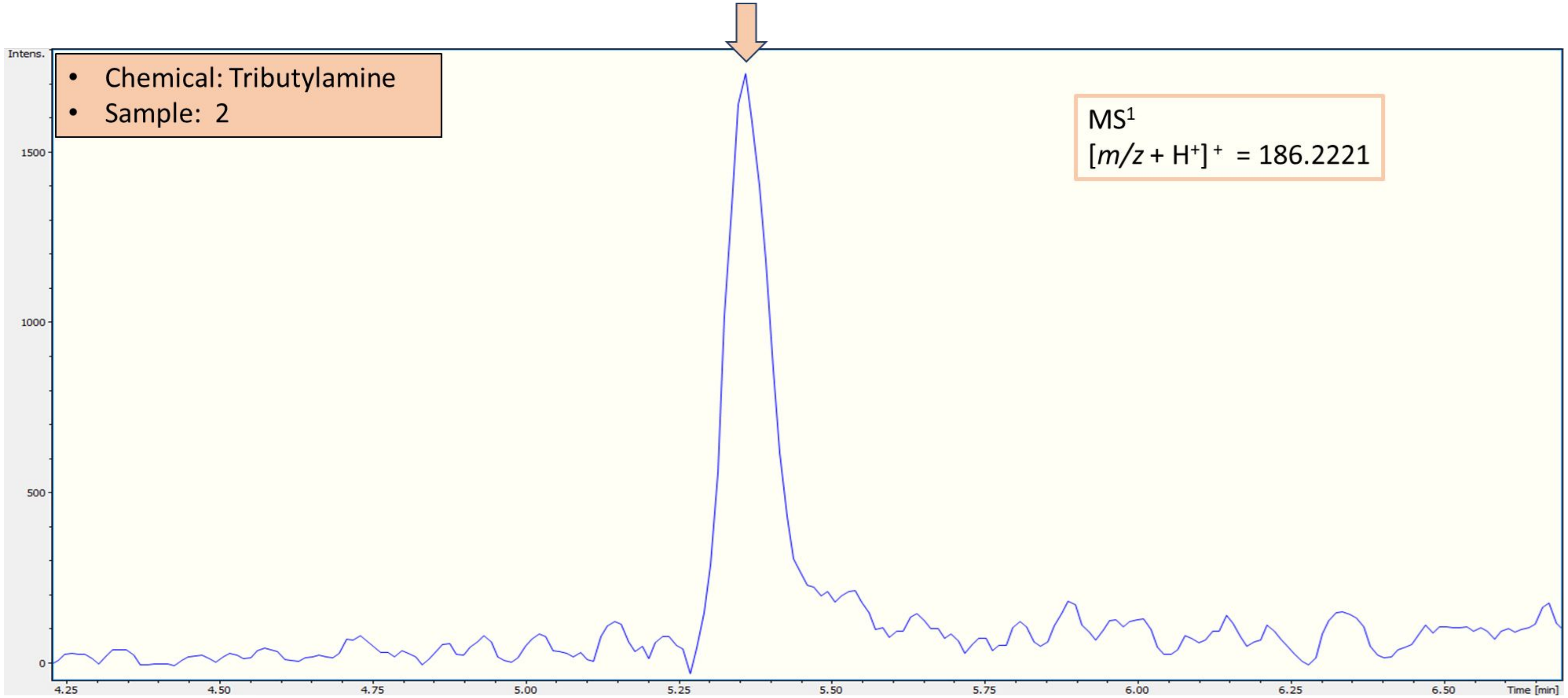
Chemical: Tributylamine
Sample: 2
MS1
[m/z + H+]+ = 186.2221
Intens.
1500
1000
500
0
4.25
4.50
4.75
5.00
5.25
5.50
5.75
6.00
6.25
6.50
Time [min]

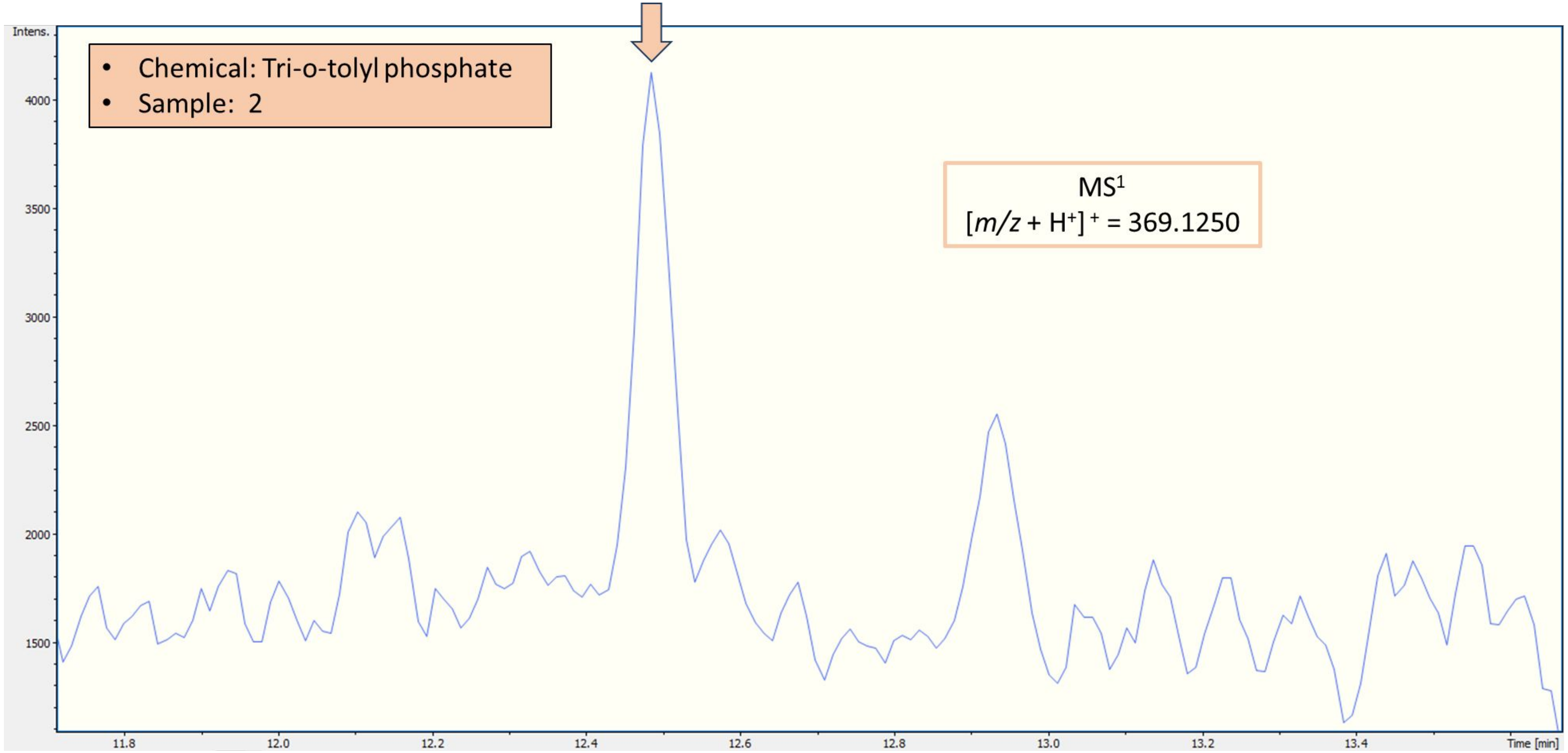
Intens.
4000
3500
3000
2500
2000
1500
11.8
12.0
12.2
12.4
12.6
12.8
13.0
13.2
13.4
Time [min]
• Chemical: Tri-o-tolyl phosphate
• Sample: 2
MS1
[m/z + H+] + = 369.1250

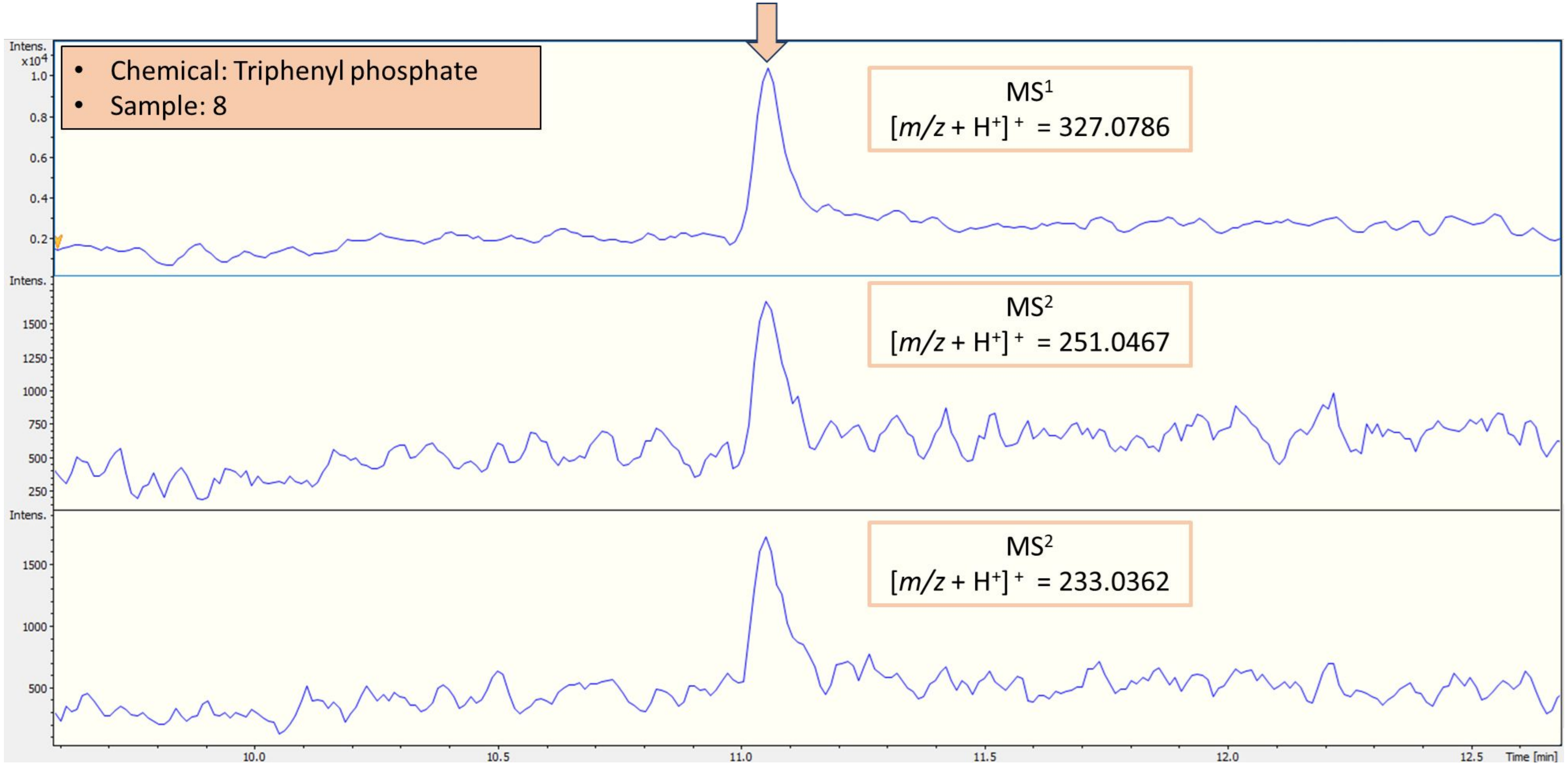

Chemical: Triphenyl phosphate
Sample: 8
MS1
[m/z + H+]+ = 327.0786
MS2
[m/z + H+]+ = 251.0467
MS2
[m/z + H+]+ = 233.0362
Intens.
x10^4
1.0
0.8
0.6
0.4
0.2
Intens.
1500
1250
1000
750
500
250
Intens.
1500
1000
500
10.0
10.5
11.0
11.5
12.0
12.5
Time [min]
